# Hypoxia associated multi-omics molecular landscape of tumor tissue in patients with hepatocellular carcinoma

**DOI:** 10.18632/aging.202723

**Published:** 2021-03-10

**Authors:** Qiangnu Zhang, Lijun Qiao, Quan Liu, Xiangpan Kong, Jun Hu, Weibin Hu, Zongze Wu, Mingyue Li, Liping Liu

**Affiliations:** 1 Department of Hepatobiliary and Pancreas Surgery, The Second Clinical Medical College, Jinan University, Shenzhen People’s Hospital, Shenzhen 518020, Guangdong, China; 2 Integrated Chinese and Western Medicine Postdoctoral Research Station, Jinan University, Guangzhou 510632, Guangdong, China; 3 Department of Hepatobiliary and Pancreas Surgery, The First Affiliated Hospital, Southern University of Science and Technology, Shenzhen 518020, China; 4 Faculty of Science and Engineering, University of Groningen, Groningen 9747, Netherlands; 5 Institute of Pathology, University Clinic of Heidelberg, Heidelberg 69120, Germany

**Keywords:** hepatocellular carcinoma, hypoxia, microenvironment, non-coding RNA, multi-omics

## Abstract

The present study was designed to update the knowledge about hypoxia-related multi-omic molecular landscape in hepatocellular carcinoma (HCC) tissues. Large-size HCC datasets from multiple centers were collected. The hypoxia exposure of tumor tissue from patients in 10 HCC cohorts was estimated using a novel HCC-specific hypoxia score system constructed in our previous study. A comprehensive bioinformatical analysis was conducted to compare hypoxia-associated multi-omic molecular features in patients with a high hypoxia score to a low hypoxia score. We found that patients with different exposure to hypoxia differed significantly in transcriptomic, genomic, epigenomic, and proteomic alterations, including differences in mRNA, microRNA (miR), and long non-coding RNA (lncRNA) expression, differences in copy number alterations (CNAs), differences in DNA methylation levels, differences in RNA alternative splicing events, and differences in protein levels. HCC survival- associated molecular events were identified. The potential correlation between molecular features related to hypoxia has also been explored, and various networks have been constructed. We revealed a particularly comprehensive hypoxia-related molecular landscape in tumor tissues that provided novel evidence and perspectives to explain the role of hypoxia in HCC. Clinically, the data obtained from the present study may enable the development of individualized treatment or management strategies for HCC patients with different levels of hypoxia exposure.

## INTRODUCTION

Hypoxia is a common microenvironmental feature of solid tumors including hepatocellular carcinoma (HCC). It leads to HCC cell proliferation activation, apoptosis inhibition, metabolic reorganization, immune escape, genetic instability, drug resistance, and angiogenesis [1, 2]. Hypoxia exposure of HCC tissues is related to the poor prognosis of patients [3]. Tumor tissue hypoxia caused by treatments such as transhepatic arterial chemotherapy and embolization may aggravate the malignant phenotype of HCC cells and impede the therapeutic effect [4, 5]. Before formulating treatment measures for hypoxia, revealing the molecular mechanism is necessary. Studies have revealed the molecular mechanism by which hypoxia plays a role in tumors [6]. However, most of this evidence was obtained from *in vitro* cell studies and animal models, while little was obtained from the tissues of HCC patients because it is not convenient to evaluate the hypoxia exposure of the tissues of the patients [7]. The molecular landscape present in hypoxia-exposed HCC tissues still lacks a comprehensive description. In order to benefit patients, especially for the development of precision medicine and individualized medicine, a large amount of tissue-level evidence is urgently needed. Bhandari reported on molecular landmarks of tumor hypoxia across 19 cancer types [8]. Based on TCGA data, Ye et al revealed hypoxia-associated molecular features to aid hypoxia-targeted therapy [9]. These studies mentioned some data about HCC, but the signature they used to assess the degree of HCC tissue hypoxia was not HCC specific, and the description of the molecular feature caused by hypoxia in HCC was not comprehensive enough. In our previous works, a novel HCC-specific hypoxia signature containing 21 stable hypoxia-related genes was constructed using mRNA expression data. Based on the 21-gene signature, we grouped patients from 10 HCC cohorts into high hypoxia exposure and low hypoxia exposure groups. Next, we comprehensively compared changes in hypoxia-related molecular features in two groups from genomic, epigenomic, transcriptomic, and proteomic perspectives to deduce the hypoxia induced molecular landscape. We believe that the molecular landscapes revealed in the present study will provide useful information for developing therapy strategies of HCC.

## RESULTS

### Transcriptomic alterations in HCC patients with different hypoxia scores

**Figure 1:**
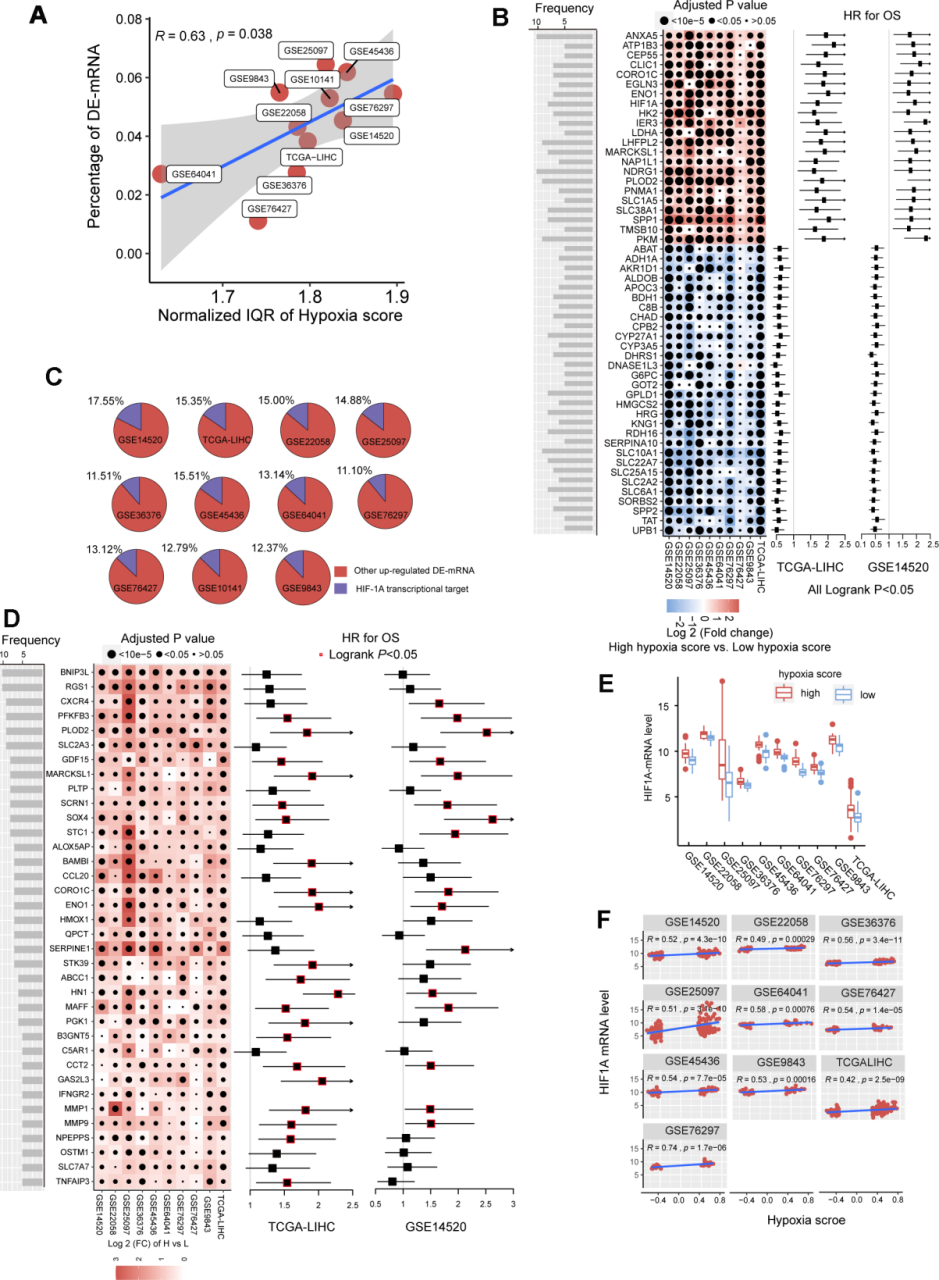
**The mRNA alterations in hepatocellular carcinoma (HCC) patients with high hypoxia scores and low hypoxia scores.** (**A**) Hypoxia scores were calculated based on the 21-gene hypoxia signature. According to the upper quartile and the lower quartile, patients were divided into a high hypoxia score group and a low hypoxia score group. In the 11 HCC cohorts, the percentage of differentially expressed (DE)-mRNAs among all mRNAs measured was positively proportional to the interquartile range (IQR) of the hypoxia scores. (**B**) A total of 51 high frequency/DE-mRNAs (HF/DE-mRNAs) are correlated to HCC patient survival in both TCGA-LIHC and GSE14520. The heat map shows the difference in the expression of these mRNAs between the high hypoxia score group and the low hypoxia score group in the 10 HCC cohorts, that is, the log2 (fold change) between the two groups. The forest plot indicates the hazard ratios (HRs) of these mRNAs for OS in the survival analysis (all logrank P < 0.01, HR < 0.7 or > 1.3, cut-off value = median expression level). (**C**) The percentage of transcription targets with differentially expressed hypoxia-inducible factor 1-alpha (HIF-1A) in a dataset for all DE-mRNAs in the dataset. (**D**) Thirty-six mRNAs may function as transcription targets of HIF-1A, and the upregulation trends are consistent in the 10 HCC datasets. The heat map shows the difference in the expression of these mRNAs between the high hypoxia score group and the low hypoxia score group. The forest plot indicates the HRs of these mRNAs for OS in the survival analysis (cut-off = median expression level). (**E**) The differences in HIF-1A mRNA expression levels between the high hypoxia score group and the low hypoxia score group in 10 HCC datasets. (**F**) Correlations between HIF-1A mRNA expression levels and hypoxia scores for the 10 HCC datasets.

In the previous studies, we established a hypoxia score system based on a novel HCC-specific 21-gene hypoxia signature that could be used to effectively estimate the hypoxia exposure in HCC tissues. In the 10 GEO datasets and the TCGA dataset, hypoxia scores of each tumor tissue samples were calculated. Patients were significantly grouped as high hypoxia exposure group and low hypoxia exposure group according to the hypoxia score (Supplementary Figure 1). First, we analyzed the differences in mRNA expression between HCC patients with high hypoxia scores (greater than the upper quartile) and those with low hypoxia scores (less than the lower quartile) cross the 11 HCC datasets. Here, mRNA with log_2_FC > 0.58 or log_2_FC <-0.58 and adjusted *P* < 0.05 were defined as differentially expressed mRNA (DE-mRNA). In these cohorts, the proportion of DE-mRNA in the total mRNA measured was positively correlated with the interquartile range (IQR) of the liver cancer tissue hypoxia scores in this cohort (Figure 1A), suggesting that the difference in mRNAs was to some extent caused by the difference in hypoxia scores. We counted the frequency of each mRNA identified as a DE-mRNA in all cohorts and included DE-mRNAs with a frequency equal to or greater than 5 in the high frequency/DE-mRNA (HF/DE-mRNA) list. The mRNA changes in this list were relatively consistent among the 10 cohorts. A total of 371 mRNAs were selected, including 192 upregulated DE-mRNAs and 179 down-regulated DE-mRNAs. For a complete HF/DE-mRNA list, see Additional File 1: Supplementary Table 2. Based on the clinical data from the TCGA-LIHC and GSE14520 datasets, we performed survival analysis on 371 HF/DE-mRNAs (logrank test, cut-off = median expression level of candidate mRNA). Through Venny analysis, we integrated the survival data from the TCGA-LIHC and GSE14520 datasets and obtained 129 HF/DE-mRNAs related to HCC survival (logrank *P*_TCGA-LIHC_ & logrank *P*_GSE14520_ < 0.05), which included 59 risk factors (*HR*_TCGA-LIHC_ & *HR*_GE14520_ > 1) and 70 protective factors (*HR*_TCGA-LIHC_ & *HR*_GE14520_ <1). There were 51 HF/DE-mRNAs that met the logrank *P* < 0.01 and *HR* < 0.7 or > 1.3 requirements in both of TCGA-LIHC and GSE14520 (Figure 1B). The results of the HF/DE-mRNA survival analysis (logrank test) are shown in Additional File 1: Supplementary Table 3.

Hypoxia-inducible factor 1-alpha (HIF-1A) plays a core role in hypoxia. 2450 potential transcription targets genes of the HIF1A transcription factor predicted using the known transcription factor binding site motifs from the TRANSFAC Predicted Transcription Factor Targets database. 10%-15% of the upregulated DE-mRNAs in each cohort may be potential transcription targets of HIF-1A (Figure 1C). Among these potential HIF-1A transcription targets, DE-mRNAs with a frequency higher than 5 among ten cohorts are shown in Figure 1D; most are risk factors for survival. Besides, although HIF-1A protein levels are known to be regulated after translation under hypoxic conditions, 10 HCC datasets indicated that the mRNA level of HIF-1A significantly increased in the high hypoxia score group and showed a significantly positive correlation with the hypoxia score (Figure 1E, 1F).

**Figure 2:**
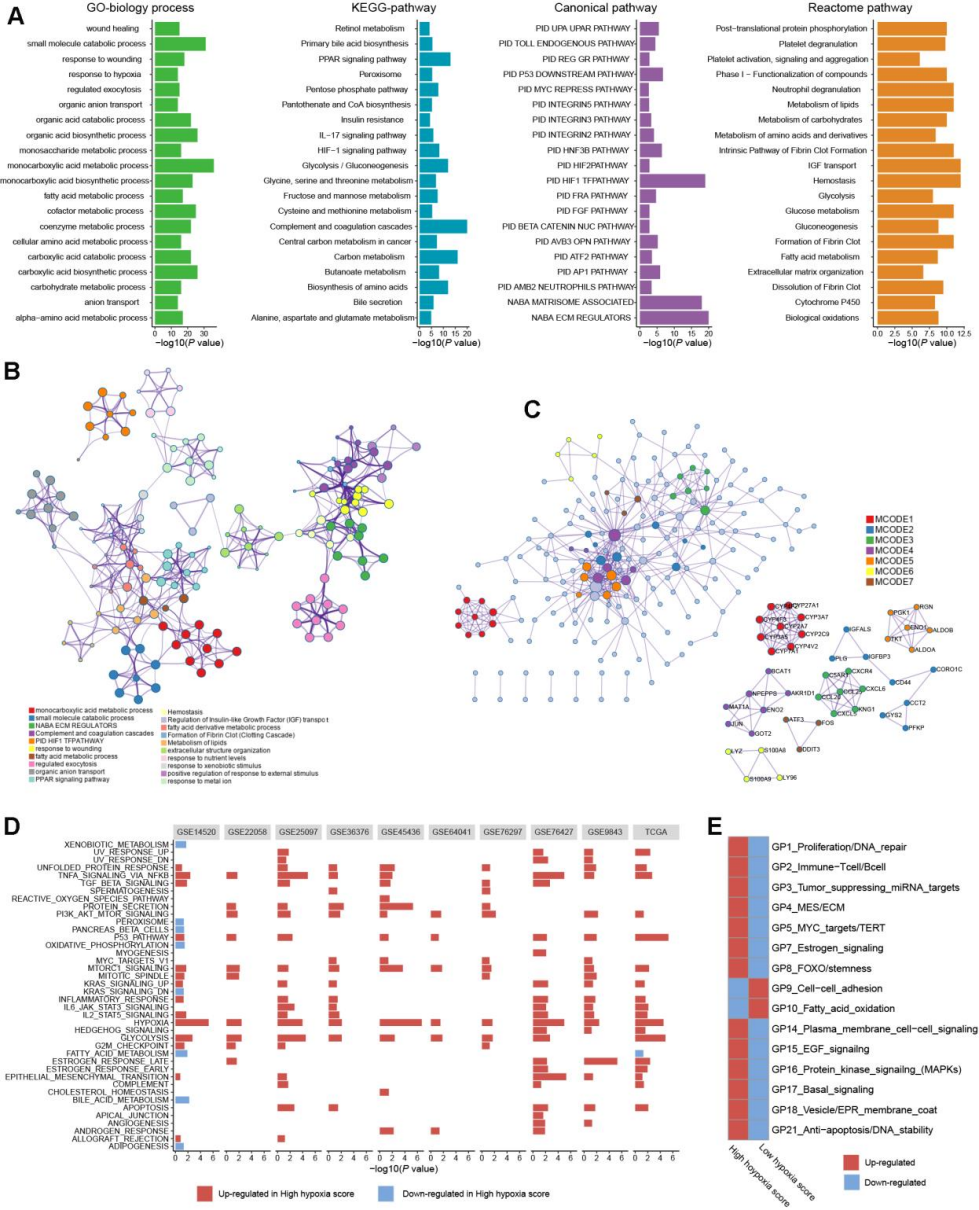
**Biological processes and pathway functional enrichment analysis of 371 high frequency/DE-mRNAs (HF/DE-mRNAs) extracted from 10 hepatocellular carcinomas (HCC) datasets.** (**A**) The top 20 (sorted by P-value) from each enrichment analysis result for GO biological processes of HF/DE-mRNAs and the pathway enrichment analysis results for 3 data sources. (**B**) A clustering network formed by correlated terms from the functional enrichment analysis. (**C**) Protein-protein interaction (PPI) enrichment network of HF/DE-mRNA translation products constructed based on the molecular complex detection (MCODE) algorithm. (**D**) Gene set enrichment analysis (GSEA) of HF/DE-mRNAs in 10 datasets, showing the pathways with P < 0.05 and false detection rate (FDR) <0.25. The reference gene sets are the hallmark gene sets. In the TCGA-LIHC dataset, (**E**) the differences between the high hypoxia score group and the low hypoxia score group in 15 gene programs related to biological behaviors of tumors. These gene programs were identified by Hoadley's team, and the upregulation and downregulation trends were calculated as single-sample GSEA z-scores.

To further reveal the functions of HF/DE-mRNAs, the enrichments of biological processes and pathways involving HF/DE-mRNA were analyzed using data from different sources, such as Gene Ontology (GO) biological processes, Kyoto Encyclopedia of Genes and Genomes (KEGG) pathways, Reactome Gene Sets, and Canonical Pathways (Figure 2A). In addition to the response to hypoxia, HF/DE-mRNAs are mainly involved in biological processes related to metabolism, including glucose metabolism, lipid metabolism, and amino acid metabolism. As expected, pathway enrichment analysis results for data from multiple sources all showed significant enrichment of the HIF-1 pathway and glucose metabolism pathways; other enriched pathways were mainly related to various metabolic pathways. Besides, some classical tumor-related pathways, such as the PI3K-AKT signaling pathway and activated protein kinase (AMPK) signaling pathway, were also significantly enriched (Additional File 1: Supplementary Table 4). The pathways related to the regulation of the extracellular matrix were also associated with HF/DE-mRNAs. Therefore, we hypothesized that hypoxia exposure might affect the extracellular matrix, which determines tumor invasion and metastasis. To find the connection between terms in the enrichment analysis of biological processes and pathways, we clustered the terms and constructed a network (Figure 2B). The name of each cluster was the name of the most representative term, and the node size was the number of genes in the term. The names of all nodes can be found in Additional File 2. For the HF/DE-mRNA translation products, protein-protein interaction (PPI) enrichment analysis was carried out with the following databases: BioGrid6, InWeb_IM7, and OmniPath8. We constructed a PPI enrichment network of physical interactions using molecular complex detection (MCODE) (Figure 2C). The names of all nodes are listed in Additional File 3. The annotation of each MCODE is shown in Additional File 1: Supplementary Table 5. In addition to focusing on HF/DE-mRNAs, we used gene set enrichment analysis (GSEA) to reveal differences between hallmark gene sets between the high hypoxia score group and the low hypoxia score group based on all mRNA differences between the two groups (Figure 2D). The hypoxia gene set was upregulated in all cohorts with high hypoxia scores. The genes composing the glycolysis gene set was upregulated in the high hypoxia score groups of 9 cohorts. Other upregulated gene sets with high consistency in the 10 cohorts included the P53 pathway, PI3K/AKT/mammalian target of rapamycin (mTOR) signaling, TNFA signaling via NFKB, unfolded protein response, TGF-beta signaling, and MTORC1 signaling. Although the fatty acid metabolism pathway was downregulated in the high hypoxia score groups in the TCGA-LIHC and GSE14520 cohorts, downregulation was not consistent among the other pathways. After applying a bimodality filter and weighted gene correlation network-based clustering, Hoadley's team identified 22 nonredundant gene programs related to the biological behaviors of tumors [10]. We found 15 gene programs that were significantly different between the high hypoxia score group and the low hypoxia score group (Figure 2E), and the single-sample GSEA z-scores for some cancer-promoting gene programs increased in patients with high hypoxia scores.

**Figure 3:**
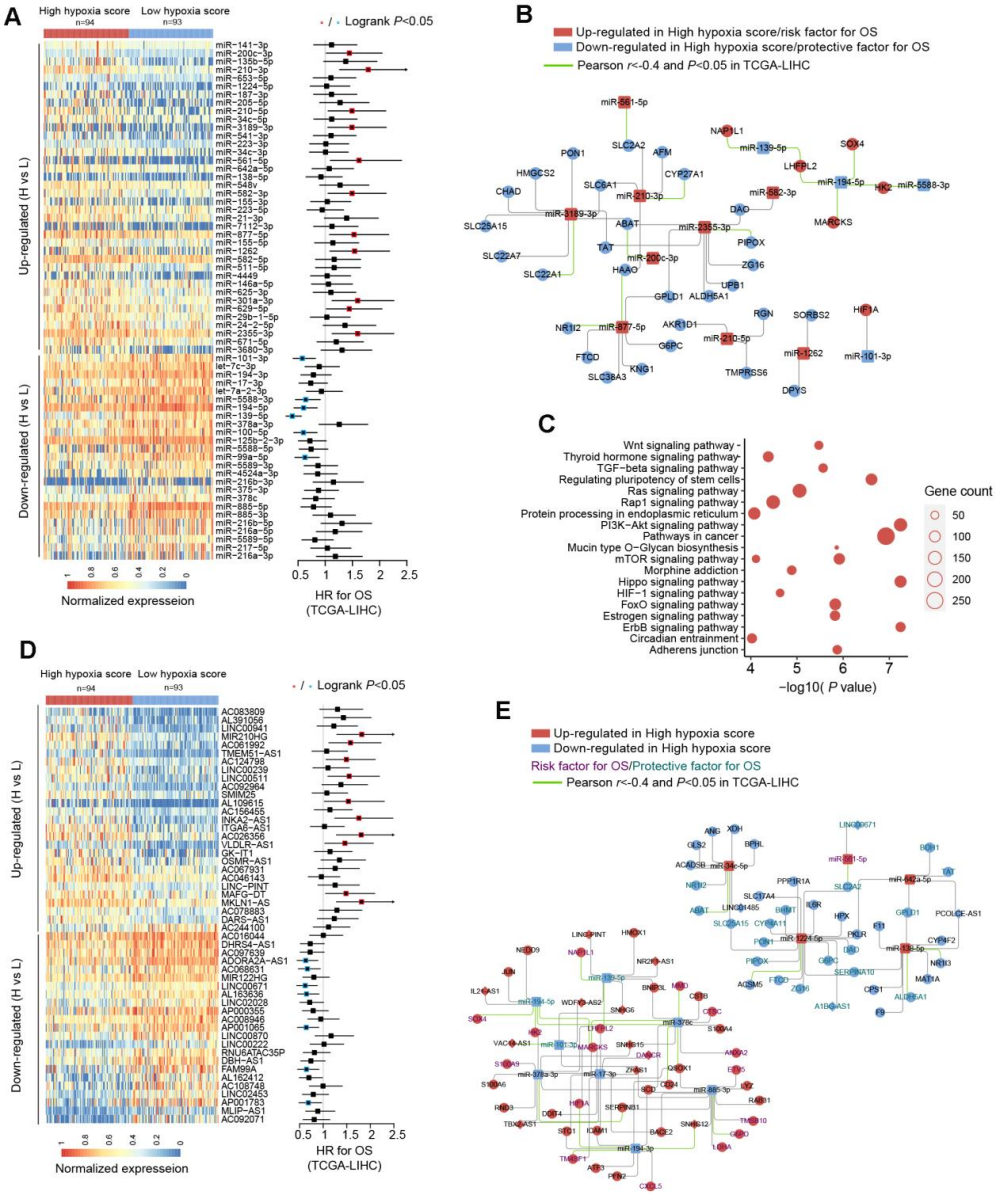
**miRNA and long non-coding RNA (lncRNA) alterations in HCC patients with high hypoxia scores and low hypoxia scores.** (**A**) A total of 63 DE-miRNAs were significantly upregulated or downregulated in the high hypoxia score group. The forest plot indicates the hazard ratios (HRs) of these miRNAs for overall survival (OS) in the survival analysis (logrank test). (**B**) Some DE-miRNAs and HF/DE-mRNAs constitute a survival-related target interaction network. All nodes in the network are correlated with HCC patient survival in TCGA-LIHC (logrank P < 0.05, cut-off = median expression level). The correlations between the nodes were calculated using Pearson correlation analysis. (**C**) Top 20 (sorted by P-value) KEGG pathway enrichment analysis results from 633 DE-miRNA target genes. (**D**) Top 50 (sorted by adjusted P-value) DE-lncRNAs that were significantly upregulated or downregulated in the high hypoxia score group. The forest plot indicates the hazard ratios (HRs) of these lncRNAs for OS in the survival analysis (logrank test). (**E**) The refined DE-lncRNA–DE-miRNA–HF/DE-mRNA ceRNA network. The correlations between nodes were calculated by Pearson correlation analysis. The survival data were from TCGA-LIHC. The cut-off is the median expression level.

We used TCGA-LIHC data to analyze the differences in microRNAs (miRs) in HCC tissues with high hypoxia scores and low hypoxia scores. The miRs with log_2_FC > 0.58 or log_2_FC < -0.58 and adjusted *P* < 0.05 were defined as differentially expressed miRs (DE-miRs). We found a total of 63 DE-miRs, including 39 upregulated DE-miRs and 24 downregulated DE-miRs. Survival analysis showed that some DE-miRs were related to the OS rate (logrank test, cut-off = median expression level) of HCC patients (Figure 3A and Additional File 1: Supplementary Table 6). In our study, miR-210-3p had the smallest adjusted *P value* among the upregulated DE-miRs (log_2_FC = 2.41). Survival analysis using the median expression level as the cut-off showed that miR-210-3p had the largest HR (logrank *P* < 0.05) and that high miR-210-3p expression indicated a poor prognosis. Additionally, the passenger strand of miR-210 (miR-210-5p) was significantly upregulated in HCC tissues with high hypoxia scores, and high miR-210-5p expression also indicated a poor prognosis. Among the downregulated DE-miRs, miR-139-5p had the smallest adjusted *P value* (log_2_FC = -0.68). The response of miR-139-5p to hypoxia has not been reported. We found that low miR-139-5p expression indicates poor outcomes of HCC patients. Next, we predicted target mRNAs of DE-miRs. Combined with the HF/DE-mRNAs list, we obtained 2 independent DE-miRs-HF/DE-mRNAs networks, including upregulated DE-miRs/downregulated DE-mRNAs and downregulated DE-miRs/upregulated DE-mRNAs (Supplementary Figure 2). HIF-1A mRNA has a targeted relationship with some downregulated miRs, such as miR-101-3p and miR-194-3p. These miRs can explain the increase in HIF-1A mRNA expression in the high hypoxia score groups of the 10 cohorts. A survival-related refined network was obtained by combining the survival analysis results (Figure 3B). In this refined DE-miR/DE-mRNA network, all nodes were associated with the survival rate of HCC patients (logrank *P* < 0.05), and a negative correlation between nodes was indicated (*r* < -0.4 and *P* < 0.05). For example, miR-194-5p showed low expression in the high hypoxia score group, which was a protective factor for the survival rate. Its potential target genes, SOX4, HK2, MARCKS, and LHFPL2, showed high expression in the high hypoxia score group and were risk factors for the OS rate. The expression of miR-194-5p was significantly negatively correlated with the mRNA expression of SOX4, HK2, MARCKS, and LHFPL2. The elements in the refined DE-miR/DE-mRNA network should receive more attention. We performed KEGG pathway enrichment analysis on all target genes of DE-miRs. The enrichment results of the top 20 (according to the P-value) were shown in Figure 3C. Some important classical pathways related to tumor development, such as the Hippo pathway, TGF-beta pathway, Ras signaling pathway, mTOR pathway, PI3K-AKT pathway, Wnt pathway, and AMPK pathway, were involved. This result further suggests that these DE-miRs may have an unignorable role in HCC tissues with high hypoxia scores. Besides, more than 80% (54/63) of the DE-miRs had at least one target gene enriched in the HIF-1 signaling pathway. This confirms that DE-miRs identified by the 21-gene hypoxia signature is indeed hypoxic-related.

Through the 21-gene signature, we revealed the presence of long non-coding RNAs (lncRNAs) that respond to and influence hypoxia exposure. In HCC tissues of patients with high hypoxia scores and low hypoxia scores, we found 719 differentially expressed lncRNAs (DE-lncRNAs), including 499 upregulated DE-lncRNAs and 220 downregulated DE-lncRNAs. The top-50 DE-lncRNAs (sorted by adjusted *P*) and survival analysis results (logrank test, cut-off = median expression level) are shown in Figure 3D. The complete lists of DE-lncRNAs and survival analysis results can be found in Additional File 1: Supplementary Tables 7, 8, respectively. miR210HG exhibited the most significant change (log_2_FC = 2.21) among the upregulated DE-lncRNAs. Higher miR210HG suggested poorer survival *(HR = 1.82,* logrank *P <* 0.05). Similarly, lncRNAs AC124798 and AC061992 were also upregulated in the high hypoxia score group and suggested poor prognosis. In addition, lncRNAs LNC00671 and FAM99A were downregulated in the high hypoxia score group, which might be protective factors for prognosis. Based on competing endogenous RNA (ceRNA) theory and expression changes, we constructed a DE-lncRNA-DE-microRNA-HF/DE-mRNA network (Figure 3E). The relationship between each node and the survival rate for HCC patients was identified, and a negative correlation between nodes was also indicated. The miR-lncRNA relationship in the network was supported by experimental evidence (provided by DIANA-LncBase). Taking lncRNA-SNHG12 as an example, it exhibits high expression in the high hypoxia score group and is a risk factor for the survival of HCC patients. lncRNA-SNHG12 and miR-194-3p may have sequence complementarity. miR-194-3p was low in the high hypoxia score group and thus is a protective factor for HCC patient survival. The target mRNAs of miR-194-3p were TM4SF1 and HIF-1A. TM4SF1 and HIF-1A showed high expression in the high hypoxia score group and thus were risk factors for HCC patient survival. TM4SF1, HIF-1A, and SNHG12 showed a significantly negative correlation with miR-194-3p. Therefore, a hypoxia-responsive lncRNA-SNHG12/miR-194-3p/TM4SF1 or HIF-1A ceRNA network is likely to present in the cancer tissues of HCC patients, and the ceRNA network is involved in tumor development and is related to patient prognosis. Leaving out sequence complementarity, we constructed the co-expression networks of all HF/DE-mRNAs, DE-miRs, and DE-lncRNAs (|Pearson r| > 0.8 and *P* < 0.05), including a positive co-network (Supplementary Figure 3A, Pearson *r* > 0.8 for all nodes) and a negative co-expression network (Supplementary Figure 3B, Pearson *r* < -0.8 for all nodes); the hub genes in the 2 networks were SERPINC1 and PKM, respectively. SERPINC1 was significantly downregulated in the high hypoxia score groups of 6 cohorts while PKM was significantly upregulated in the high hypoxia score groups of 10 cohorts (Supplementary Figure 3C). Compared with those in normal tissues, SERPINC1 was significantly lower and PKM was significantly higher in HCC tissues (Supplementary Figure 3D). Combined with the survival analysis results (Supplementary Figure 3E), we speculated that SERPINC1 and PKM play important roles, namely, cancer-suppressing and cancer-promoting functions, respectively, under hypoxia exposure. Supplementary Figure 4 displays all mRNAs and lncRNAs related to HIF-1A mRNA levels (|Pearson r|> 0.4 and *P* < 0.05), and the relationship between nodes and survival was indicated. Among miRs, only microRNA-194-3p and microRNA-194-5p had a significantly negative correlation with HIF-1A. These data may help to explain the differences in HIF-1A mRNA between groups with high hypoxia scores and reflect the core role of HIF-1A mRNA.

### Genomic alterations in HCC patients with different hypoxia scores

**Figure 4:**
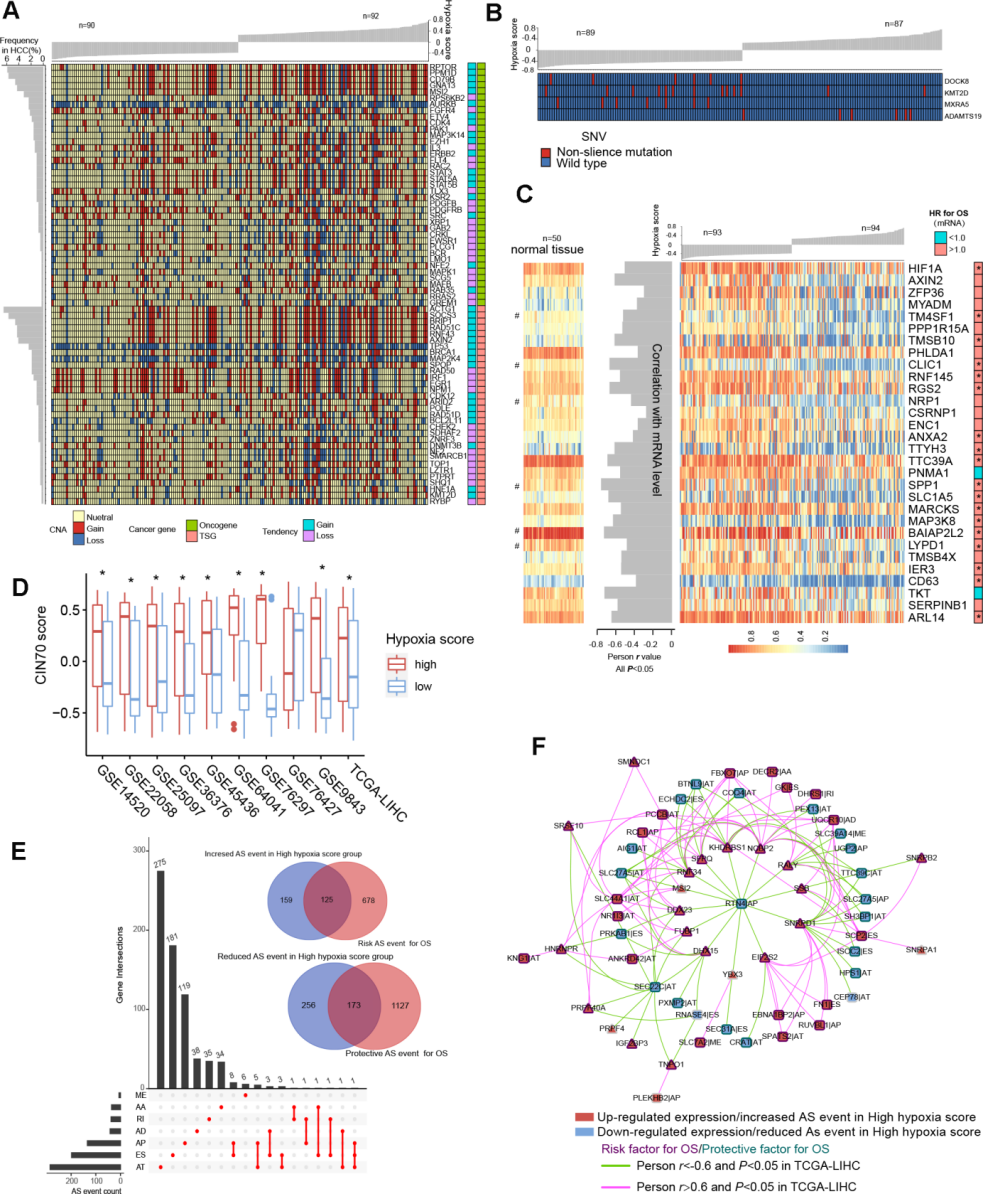
**Differences in genomic and epigenetic alterations between groups with high hypoxia scores and low hypoxia scores.** (**A**) The difference in the incidence of copy-number aberrations (CNAs) in 71 cancer genes between the high hypoxia score group and the low hypoxia score group. (**B**) The proportions of single-nucleotide variants (SNVs) in 4 genes are significantly different between the high hypoxia score group and the low hypoxia score group. (**C**) Reductions in the methylation levels of 30 genes are accompanied by significant increases in the corresponding mRNA levels in the high hypoxia score group. The correlation between DNA methylation and corresponding mRNA expression was obtained through Pearson correlation analysis based on TCGA-LIHC data. The hazard ratios (HR) of the corresponding mRNAs for overall survival (OS) were calculated by the logrank test for TCGA-LIHC data, and the cut-off was the median expression level. (**D**) 70-gene chromosome instability (CIN70) was used to assess chromosome instability in tumor tissues from 10 hepatocellular carcinomas (HCC) datasets. The CIN70 scores are significantly different between tumor tissues with high hypoxia scores and low hypoxia scores. (**E**) The occurrences of 713 AS events are significantly different between the high hypoxia score group and the low hypoxia score group. Some of the AS events are associated with the OS of HCC patients. (**F**) The expression of 30 splicing factors in the high hypoxia score group and the low hypoxia score group are different, and their expression trends are consistent in 10 datasets. These splicing factors and the AS events with different occurrences between the two groups form a network. The correlations between the nodes of the network were calculated by Pearson correlation analysis based on TCGA-LIHC data. The relationship between the nodes and the OS of HCC patients was obtained through univariate cox survival analysis.

TCGA-LIHC data were used to reveal somatic copy number aberrations (CNAs) and somatic single-nucleotide variants (SNVs) in HCC patients with different hypoxia scores. First, from the overall differences in gene-level CNAs in patients with high hypoxia scores (greater than the upper quartile) and low hypoxia scores (less than the lower quartile), we found that CNAs of approximately 13.7% (3396/24769) of the genes were concentrated in the high hypoxia score group (Supplementary Figure 5A and Additional File 1: Supplementary Table 9). CNA events in 71 cancer genes (according to the definition of the Precision Oncology Knowledge Base (OncoKB) cancer gene list) were significantly differentially distributed between the two groups (Figure 4A). The copy number gain to copy number loss ratios for most genes were significantly increased in the high hypoxia score group. The roles and CNA tendencies (according to the proportions of homozygous deletions, single copy deletions, low-level copy number amplification, and high-level copy number amplification) of these 71 cancer genes were provided.

For example, CDK4 is an oncogene, and its CNAs in the high hypoxia score group are mostly copy number gains. IRF1 is a tumor suppressor gene, and its CNAs in the high hypoxia score group were mostly copy number losses. The occurrence frequency of CNAs of some cancer genes was high in patients in the TCGA-LIHC cohort. Next, we analyzed the difference in gene-level SNVs between the two groups of patients. Unfortunately, we did not obtain much evidence that indicated a strong connection between SNVs and hypoxia score. Only 4 genes were significantly different in the incidence of SNVs between the two groups (Figure 4B). Among them, the proportion of non-silent mutations only increased in ADAMTS19 in the high hypoxia score group. Besides, we found that SNVs in 172 genes tended to be concentrated in the low hypoxia score group or the high hypoxia score group (Supplementary Figure 5B). However, because the overall mutation frequency of these genes was not high, there was no statistically significant difference in the distribution of SNVs between the two groups.

### Epigenetic alterations in HCC patients with different hypoxia scores

We found that there were significant differences in the methylation levels at 464 gene loci between patients with high hypoxia scores and patients with low hypoxia scores and that the methylation levels at most loci were significantly reduced in the high hypoxia score group (Supplementary Figure 5C and Additional File 1: Supplementary Table 10). The methylation level increased at only a few loci in the high hypoxia score group. We jointly analyzed methylation levels and mRNA expression levels and found a significant increase in mRNA expression levels of 30 genes in the high hypoxia score group (data from TCGA-LIHC) and a simultaneous decrease in their methylation levels (Figure 4C). The mRNA expression levels of these genes showed a significantly negative correlation with their methylation levels, and the high mRNA expression of most of these genes is a risk factor for HCC patient survival. The methylation loci corresponding to these 30 genes and their distance from the transcription start site (TSS) are shown in Additional File 1: Supplementary Table 11. Notably, the degree of methylation of HIF-1A was also reduced in the high hypoxia score group, which provides another explanation for the increase in HIF-1A mRNA expression in the high hypoxia score group. From the comparison of DNA methylation levels of the 30 genes in normal tissues and HCC tissues, the DNA methylation levels of some genes in normal tissues were not different from those in HCC tissues with low hypoxia scores but were higher than those in HCC tissues with high hypoxia scores (such as RGS2), and the DNA methylation levels of some other genes were highest in normal tissues, followed by those in HCC tissues with low hypoxia scores, and lowest in HCC tissues with high hypoxia scores (such as CLIC1).

Chromosome instability occurs throughout the development and progression of tumors. The increase in chromosome instability will lead to HCC cell growth and enhanced invasiveness [11]. The 70-gene chromosome instability (CIN70) signature constructed by Carter et al. can effectively assess the chromosome instability of tumor cells [12]. We compared the CIN70 scores between patients with high hypoxia scores and patients with low hypoxia scores. The data showed that in 9 datasets, the CIN70 scores were significantly increased in the high hypoxia score groups (Figure 4D), suggesting that hypoxia exposure may cause chromosome instability in HCC cells.

Alternative splicing (AS) is an epigenetic feature that plays an important role in tumorigenesis and development [13]. Based on the TCGA-LIHC data, we found that the occurrences of 713 AS events (involving 681 mRNAs) differed significantly between the two groups and that most of these events were alternative terminator (AT), exon skipping (ES), and alternative promoter (AP) events (Figure 4E). In the high hypoxia score group, the percent spliced in (PSI) value of retained intron (RI) events of EPHB2 mRNA had the maximum reduction (adjusted *P* value < 0.001), and the AT events of GULP1 mRNA had the most increased PSI (adjusted *P* value < 0.01). The Cox analysis results suggested that the AS events with differences in occurrence between the two groups were related to HCC patient survival. Detailed information on these differentials AS events is provided in Additional File 1: Supplementary Table 12. The 681 mRNAs involved in differential AS events were subjected to functional enrichment analysis of biological processes and pathways. In addition to the HIF1 pathway and sugar and lipid metabolism pathways (indicating their relations to hypoxia exposure), these mRNAs are also involved in various classical pathways of tumorigenesis and development (Supplementary Figure 6A). The PPI network of these mRNA translation products is shown in Supplementary Figure 6B. In addition, we analyzed the differences in expression of 404 splicing factors (SFs) between the high hypoxia score group and the low hypoxia score group. The differences in expression of 30 SFs were relatively consistent in 10 datasets (the condition of adjusted *P* < 0.05 was met in 5 or more datasets). Except for NRIP2, other SFs were upregulated in the high hypoxia score group (Supplementary Figure 7A). Next, correlations among these SFs and 713 AS events were analyzed. When constructing the correlation network, we only set the thresholds at |Person r |> 0.6 and *P* < 0.05 and did not perform other node screening. Notably, in the obtained correlation network, each node reached logical harmony in variation trend and prognostic value (Figure 4F). For example, KHDRBS1 is an SF with elevated expression in the high hypoxia score group and is a risk factor for HCC patient survival. The occurrences of 7 AS events that were negatively correlated with KHDRBS1 were reduced in the high hypoxia score group, and these 7 AS events were protective factors for HCC patient survival. In contrast, 5 AS events that were positively correlated with KHDRBS1 increased in the high hypoxia score group, and these AS events were risk factors for HCC patient survival. Based on the evidence, we believe that AS events may be a response to hypoxia in HCC cells. Some hypoxia-induced changes in cell functions and behaviors may be achieved through the SF-AS network. The difference in AS between the high hypoxia score group and the low hypoxia score group may explain the prognosis difference between the two groups.

RNA N6-methyladenosine (m^6^A) modification is another important epigenetic feature of RNA [14]. The expression differences in 21 m^6^A regulators between the high hypoxia score group and the low hypoxia score group were analyzed. In the 10 HCC datasets, most m^6^A regulators showed no significant difference between the two groups (Supplementary Figure 7B), but 1 m^6^A writer (WTAP), 1 m^6^A eraser (ALKBH5), and 1 m^6^A reader (YTHDF2) significantly increased in the high hypoxia score groups of 4 or more datasets.

### Changes in functional proteomics in HCC patients with different hypoxia scores

**Figure 5:**
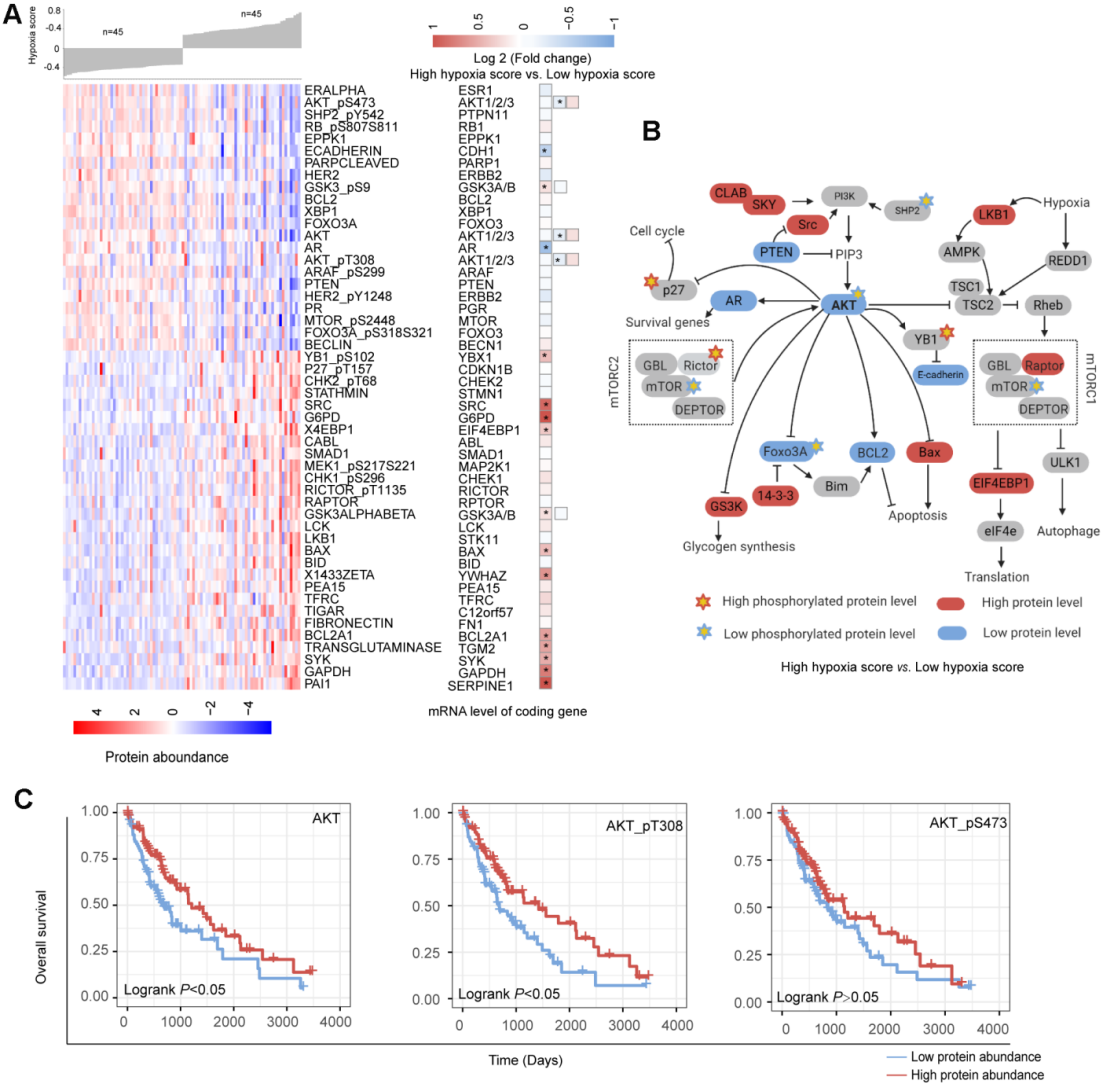
**Changes in functional proteomics between hepatocellular carcinoma (HCC) patients with high hypoxia scores and low hypoxia scores.** (**A**) Fifty proteins with significant differences in abundance between HCC patients with high hypoxia scores and low hypoxia scores. (**B**) A schematic diagram of some proteins with significant differences in abundance in the AKT/mTOR pathway. (**C**) The low abundance of AKT, AKT-pT308, and AKT_pS473 proteins in TCGA-LIHC indicates poor overall survival.

Based on the reverse phase protein array (RPPA) data of the TCGA-LIHC cohort, we analyzed the differences in the abundance of 218 proteins between the high hypoxia score group and the low hypoxia score group. A total of 50 proteins exhibited differences in abundance between the two groups (Figure 5A). Increases in LKB1 and mTOR activity inhibition are hallmark events of hypoxia exposure, which once again confirmed the efficacy of our 21-gene signature in indicating hypoxia exposure. Approximately half of the protein changes were inconsistent with the changes in their mRNA levels, suggesting that the regulation of posttranslational levels by hypoxia cannot be ignored. The differentially expressed proteins were mainly concentrated in AKT/mTOR and its related pathways. The reduction in the AKT level and its phosphorylation level seems to be a core event. The change trends for some proteins were opposite to those reported by the relevant literature on AKT (Figure 5B), which suggests that in addition to the regulation of AKT, there may be a complex regulatory network that has not been discovered. Survival analysis (cut-off = median value, Figure 5C) showed that a low abundance of AKT, AKT-pT308 and AKT_pS473 indicated worse OS (except for AKT_pS473, logrank *P* < 0.05). Hypoxia may induce some functions by inhibiting AKT in HCC. Overall, the changes in the proteins between the two groups reflected the dynamic balance of autophagy/apoptosis and the response to hypoxic stress to some extent. These proteins may affect the viability of the cells under hypoxia and their tolerance to hypoxia. Moreover, the altered protein abundance between the two groups may be related to the different drug sensitivities of HCC patients in the two groups. The correlation between some proteins differentially expressed in the two groups and drug sensitivity is shown in Supplementary Figure 8 (based on data from the MD Anderson Cell Lines Project and the Genomics of Drug Sensitivity in Cancer database). For example, the reduction in AKT_pS473 may cause the sensitivity of cells to sorafenib to decrease. Therefore, when formulating a treatment plan, it may be necessary to evaluate the hypoxic condition of the lesions.

## DISCUSSION

The molecular changes in cancer cells caused by hypoxia have been widely reported in studies *in vitro* [15, 16]. However, the molecular changes caused by hypoxia in tissues may be different from the data obtained *in vitro* [17]. If it is necessary for patients to truly benefit from anti-hypoxic treatment strategies, only the data obtained from the cellular hypoxia model is far from enough. Polarographic electrodes can accurately measure the degree of hypoxia exposure in cancer tissues, but this method does not have enough practicality [18]. Hypoxia can change the gene expression level in tumor cells. Therefore, the detection of the levels of specific genes can indirectly reflect hypoxia exposure in tissues [19]. In the past decade, different hypoxia gene signatures have been reported, and some excellent signatures, such as Buffa's 15-gene hypoxia signature, have proven to have the ability to indicate hypoxia in a variety of tumors [20]. Sorensen reported a group of hypoxia gene markers indicating the hypoxia in human squamous cell carcinomas [21]. Inspired by these studies we have identified a novel 21-gene hypoxia signature that has excellent robustness in the assessment of hypoxia exposure. This provides a basis for us to explore the hypoxia-induced molecular landscape in HCC. After calculating hypoxia scores using the 21-gene hypoxia signature, we found that the hypoxia scores in the tumor tissues of HCC patients in 11 datasets were grouped into 2 clusters with different hypoxia exposure levels. Because the number of probes in the GSE10141 dataset was small, this dataset was excluded in subsequent studies. Considering the numerical distribution of hypoxia scores in tissues, we selected the upper quartile and the lower quartile to divide high hypoxia scores and low hypoxia scores. In this way, more differential changes may be obtained, creating a situation that is more in line with clinical practice. Because hypoxic conditions and molecular changes in tumor tissues are nonlinearly correlated, mild hypoxia exposure may not cause many molecular events. After comprehensive analysis in the present study, the patients in the high hypoxia score group and the patients in the low hypoxia score group were found to have many transcriptomic, genomic, epigenomic, and proteomic differences.

Among HF/DE-mRNAs, all risk factors for OS were significantly upregulated in the high hypoxia score group while the protective factors were downregulated; this phenomenon occurred in almost all cohorts. For example, ANXA5 and SPP1 were both risk factors for OS in the TCGA-LIHC and GSE14520 datasets and were significantly overexpressed in the high hypoxia score group in 9 cohorts. The results of pathway enrichment analysis indicated the P53 pathway, PI3K/AKT, (mTOR) signaling, and TGF-beta signaling upregulated in the high hypoxia group. The activation of these pathways is critical to the onset of the progression of tumors and can be used to explain the poor prognosis of patients in the high hypoxia score group. In addition, many drugs target these pathways, suggesting that the degree of hypoxia exposure needs to be considered in the selection of medications for patients with HCC. Among DE-miRs, miR-210 is a frequently reported master hypoxia-responsive miR, and its high expression has been observed in a variety of hypoxia-treated tumor cells [22, 23]. The presence of miR-210 in the DE-miR list means that our 21-gene hypoxia signature can reflect hypoxia exposure in HCC. We also obtained DE-miRs-HF/DE-mRNAs networks. These networks suggested that the changes in some DE-mRNAs in HCC tissue under hypoxic conditions might be caused by the targeted regulation of DE-miRs. For example, miR-216b-5p is the miR with the highest degree of reduced expression in the high hypoxia score group (log2FC = -2.61, adjusted P < 0.001), and it is complementary to the 3' untranslated region (3'-UTR) of HK2. The prediction was supported by argonaute-crosslinking immunoprecipitation sequencing (AGO-CLIP-seq) data [24, 25]. HK2 is a definite hypoxia-inducible gene [26]. The above results suggest that the high expression of HK2 in hypoxia may be associated with the reduction in miR-216b-5p. These 2 networks provide additional explanations for hypoxia-induced transcriptome changes in HCC tissues in addition to the HIF-1A-related mechanism. Attention needs to be paid to miRs with high connectivity in the network, such as miR-1224-5p, miR-877-5p, let-7a-2-3p, and miR-378c. DE-lncRNA was also analyzed as well as mRNAs and miRs. Moreover, we constructed a DE-lncRNA-DE-microRNA-HF/DE-mRNA network. There were differences in the expression of mRNAs, miRs, and lncRNAs between the high hypoxia score and low hypoxia score groups. Many cancer-promoting RNA molecules were upregulated in the high hypoxia score group while cancer-suppressing RNA molecules were downregulated in the high hypoxia score group. Some DE-mRNAs and/or DE-lncRNAs and DE-mRNAs may form regulatory networks that participate in the development of HCC and affect the prognosis of HCC patients.

In genomic alterations analysis, CNAs changes suggested that the genomic instability of HCC patients may be related to hypoxia exposure. Few genes were significantly different in the incidence of SNVs between the high hypoxia and low hypoxia score groups. Hence, we believed that hypoxia has a limited ability to induce SNVs, and important SNVs likely occur before the onset of hypoxia. Epigenetic alterations suggested that hypoxia exposure may promote the hypomethylation of some genes related to the onset and progression of HCC and that the hypomethylation of some other genes may be a characteristic feature of hypoxia exposure. Moreover, RNA alternative splicing and m6A modification were another important epigenetic feature under hypoxia which expanded our understanding of the role of hypoxia in HCC.

Some differential molecular events identified in the present study are closely related to the prognosis of patients and are molecular mechanisms that explain the cancer-promoting effect of hypoxia. Therefore, these differential events can help screen HCC diagnostic and treatment targets. The results at multi-omic levels had some consistency, such as the simultaneous presence of high mRNA expression and decreased methylation of the corresponding DNA in the high hypoxia score group. However, some results were contradictory. For example, the GSEA results for the AKT/mTOR pathway based on the mRNA level were not consistent with the proteomic results for the AKT/mTOR pathway. Therefore, it is necessary to investigate the molecular changes caused by hypoxia using multi-omics approaches. Based on the available data, we analyzed the relationships between differential events and patient prognosis and extracted useful data for translational medicine. In the Supplementary Files, we provided reference data with details, such as the lists of DE-mRNAs, DE-miRNAs, and DE-lncRNAs, the list of genes with CNAs, the list of genes with aberrant methylation loci, and the list of hypoxia-related RNA alternative splicing events. We hope that these data can inspire and help other researchers, improve research efficiency, and narrow the scope of research.

It should be emphasized that we found that the HIF-1A mRNA levels significantly increased in the high hypoxia score groups in the 10 datasets and showed a significantly positive correlation with the hypoxia score. The reason why this point is emphasized is that most previous studies have focused on the posttranslational regulation of HIF-1A at the protein level, and some researchers even deliberately ignore changes in HIF-1A mRNA [27]. Our data suggest that the regulation of HIF-1A mRNA levels cannot be ignored in hypoxia exposure, meaning that in treatment regimens targeting HIF-1A, not only HIF-1A protein but also HIF-1A mRNA should be targeted. In addition, our data showed that during hypoxia exposure, the level of HIF-1A might be regulated by a multilevel positive feedback network and that an mRNA/lncRNA network and methylation regulation might be part of this network.

In summary, the present study provided tissue-level evidence for throwing light on the mechanism by which hypoxia may perform its function in HCC. An unprecedented comprehensive panorama of hypoxia associated multi-omics molecular features was systematically described. Networks between molecular features were tried to establish such as DE-lncRNA-DE-microRNA-HF/DE-mRNA networks. Clinically, the data obtained from this study may facilitate developing possible treatment or diagnostic novel targets under the perspective of personalized medicine.

## MATERIALS AND METHODS

### Public datasets in GEO

We retrieved three independent mRNA microarray datasets (GSE18494, GSE55214, and GSE57613) based on hypoxia treated HCC cells from the Gene Expression Omnibus (GEO, https://www.ncbi.nlm.nih.gov/geo/). Datasets and available clinical information of ten HCC cohorts (GSE14520, GSE22058, GSE25097, GSE36376, GSE45436, GSE64041, GSE76297, GSE76427, GSE10141 and GSE9843) were downloaded from GEO. All gene symbols in GEO datasets were converted to the latest HGNC (HUGO Gene Nomenclature Committee) symbols.

### Multi-omic data and clinical data of TCGA-LIHC

mRNA expression, microRNA expression, lncRNA expression, CNAs, SNVs, DNA methylation data of the Cancer Genome Atlas Liver Hepatocellular Carcinoma (TCGA-LIHC) were downloaded from the TCGA data portal (https://portal.gdc.cancer.gov/). The RNA alternative splicing data of TCGA-LIHC were obtained from TCGA SpliceSeq (http://projects.insilico.us.com/TCGASpliceSeq/). The reverse phase protein array (RPPA) data of TCGA-LIHC were downloaded from the cancer proteome atlas (https://www.tcpaportal.org/tcpa/index.html).

### Hypoxia scores calculation

In our previous study, a novel 21-gene hypoxia signature was built using robust rank aggregation (RRA) algorithm from the microarray data of hypoxia treated HUH7, SNU-182, and HLF cells. Hypoxia score was calculated using gene set variation analysis (GSVA) based on the 21 genes. The gene lists of 21-gene signature were shown in Additional File 1: Supplementary Table 1.

### Biological process and pathway enrichment assay

The biological process and pathway enrichment assay of candidate genes were performed using online tools provided by Metascape (http://metascape.org/gp/index.html). The enrichment analysis has been carried out with the following ontology sources: the Kyoto Encyclopedia of Genes and Genomes (KEGG) Pathway, Gene Ontology (GO) biological processes, Reactome Gene Sets, and Canonical Pathways All genes in the genome have been used as the enrichment background. To establish the network based on the relationships between the terms, a subset of enriched terms with a similarity > 0.3 were connected by edges. 20 clusters were obtained and the terms with the best p-values were selected (for more details see the website of Metascape). The network was visualized using Cytoscape (version 3.7.2).

### Protein-protein interaction enrichment analysis

We used online tools provided by Metascape to perform protein-protein interaction enrichment analysis for the production of candidate genes. According to data from BioGrid, InWeb, and OmniPath, a resultant network contains the subset of proteins that form physical interactions with at least one other member was built using Molecular Complex Detection (MCODE) algorithm [28]. Then pathway and process enrichment analysis has been applied to each MCODE component (for more details see the website of Metascape). The network was visualized using Cytoscape (version 3.7.2).

### Gene set enrichment analysis (GSEA)

GSEA was performed for candidate mRNAs across HCC cohorts using GSEA tools (version 4.0.3) provided by the Molecular Signatures Database (MSigDB, https://www.gsea-msigdb.org/gsea/msigdb/index.jsp). The hallmark gene was set as the reference genes. The significantly activated or suppressed pathways were identified as pathways with *P* value<0.05 and FDR<0.25.

### Interactions analysis for mRNA/microRNA/lncRNA

miRNA-target interactions were presented by intersecting the predicting target sites of miRNAs with binding sites of Ago protein using the Encyclopedia of RNA Interactomes (ENCORI, http://starbase.sysu.edu.cn/index.php) and miRwalk 3.0 (http://zmf.umm.uni-heidelberg.de/apps/zmf/mirwalk/). The miRNA-lncRNA interactions were presented using LncBase v.2 experimental module tools (http://diana.imis.athena-innovation.gr/DianaTools/index.php). Interactions of mRNA/microRNA/lncRNA were visualized using Cytoscape (version 3.7.2).

### Statistical analysis

Statistical analyzes were performed using R software (version 3.6.1) with relevant packages. In brief, the differential expressed mRNAs were extracted from microarray datasets using the Limma package. The differential expressed mRNAs, microRNA, and lncRNA of TCGA-LIHC were identified using Linnorm packages. The difference between the two groups was compared using the independent *t*-test or Wilcox test. The adjusted *P* value was obtained using the false discovery rate (FDR) method. Coefficients were calculated using Pearson or Spearman’s correlation analysis. A chi-squared test was used to determine the significant difference between the frequencies. Survival analysis was performed using Univariate Cox/multivariate analysis hazard analysis or Kaplan-Meier survival estimate using the survival package. The forest-plot R package was employed to visualize the hazard rate obtained from survival analysis. The Kaplan-Meier survival curves were created using the survminer package with the logrank test. In the present study, statistical significance was set at a probability value of *P* < 0.05.

## Supplementary Material

Supplementary Figures

Supplementary Table 1

Supplementary Table 2

Supplementary Table 3

Supplementary Table 4

Supplementary Tables 5 and 6

Supplementary Table 7

Supplementary Table 8

Supplementary Table 9

Supplementary Table 10

Supplementary Table 11

Supplementary Table 12
