# Supplementary material for: Hypoxia associated multi-omics molecular landscape of tumor tissue in patients with hepatocellular carcinoma
Source: Aging (Albany NY). 2021 Mar 10;13(5):6525–53. doi: 10.18632/aging.202723 (PMC7993683; doi:10.18632/aging.202723)
Supplement: Supplementary Figures [file aging-13-202723-s001.pdf]

SUPPLEMENTARY FIGURES

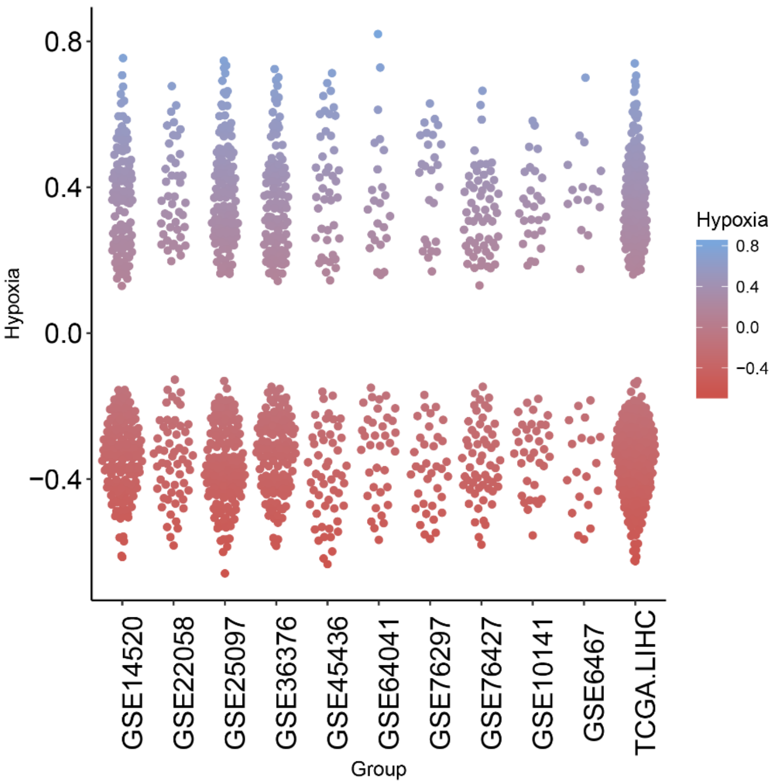

Supplementary Figure 1. The distribution of hypoxia scores calculated based on the 21-gene hypoxia signature in the cancer tissues of 11 HCC cohorts.

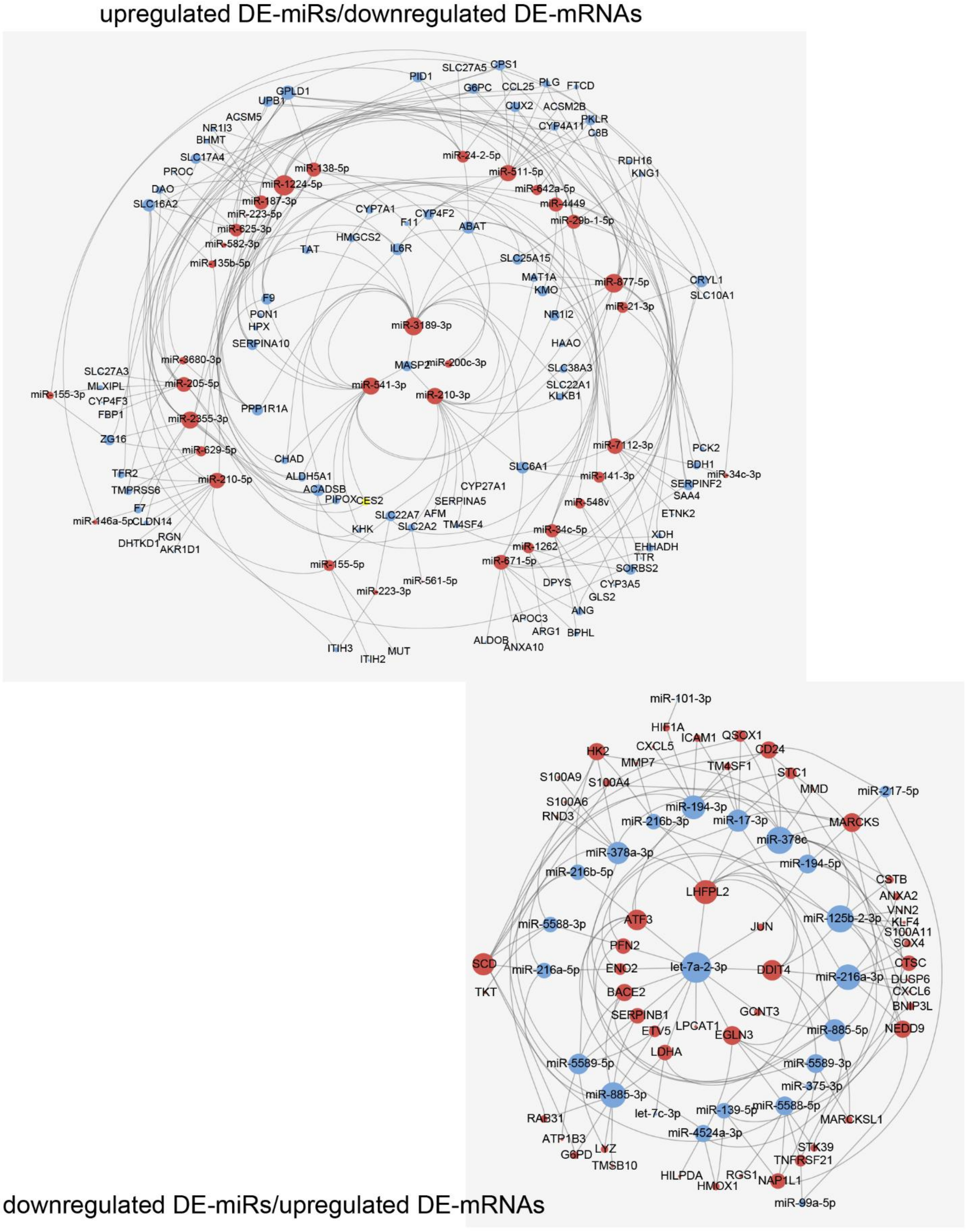

Supplementary Figure 2. DE-miRNAs and HF/DE-mRNAs form a target interaction network. Target interactions were determined by intersecting the predicted target sites of miRNAs with binding sites of Argonaute protein using the online tools ENCORI and miRwalk 3.0.

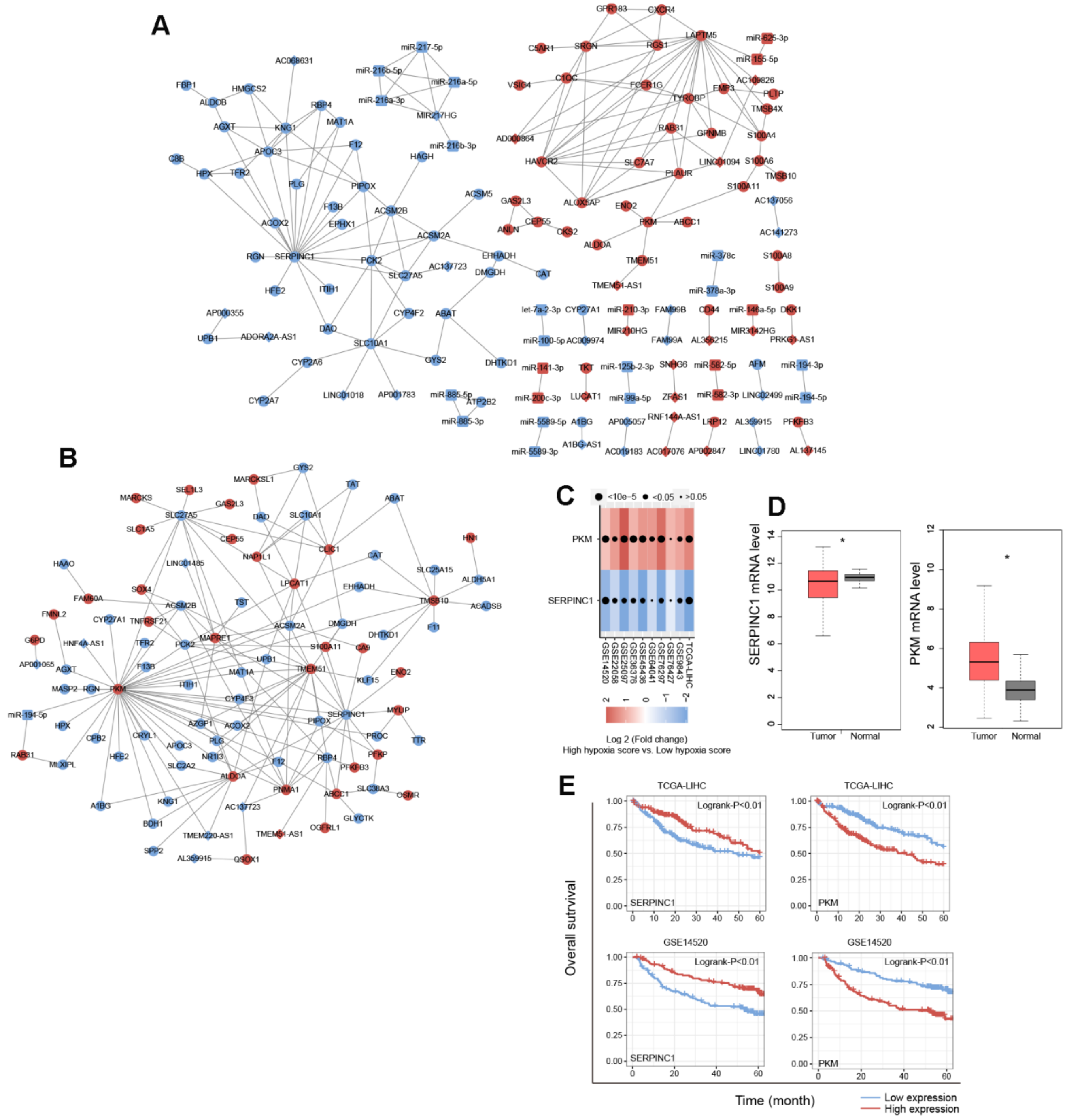

Supplementary Figure 3. A co-expression network formed by HF/DE-mRNAs, DE-miRs, and DE-lncRNAs. (**A**, **B**) The correlations among HF/DE-mRNAs, DE-miRs, and DE-lncRNAs were obtained using Pearson correlation analysis. The nodes with |Pearson r| > 0.8 and P < 0.05 constitute positive and negative co-expression networks. (**C**) In the positive and negative co-expression networks, the 2 genes with the highest connectivity are SERPINC1 and PKM. Their differences among 10 hepatocellular carcinomas (HCC) datasets are shown in the heat map. (**D**) Differences in the expression of SERPINC1 and PKM between normal tissues and cancer tissues of HCC patients in TCGA-LIHC. (**E**) SERPINC1 and PKM are associated with patient survival in TCGA-LIHC and GSE14520.

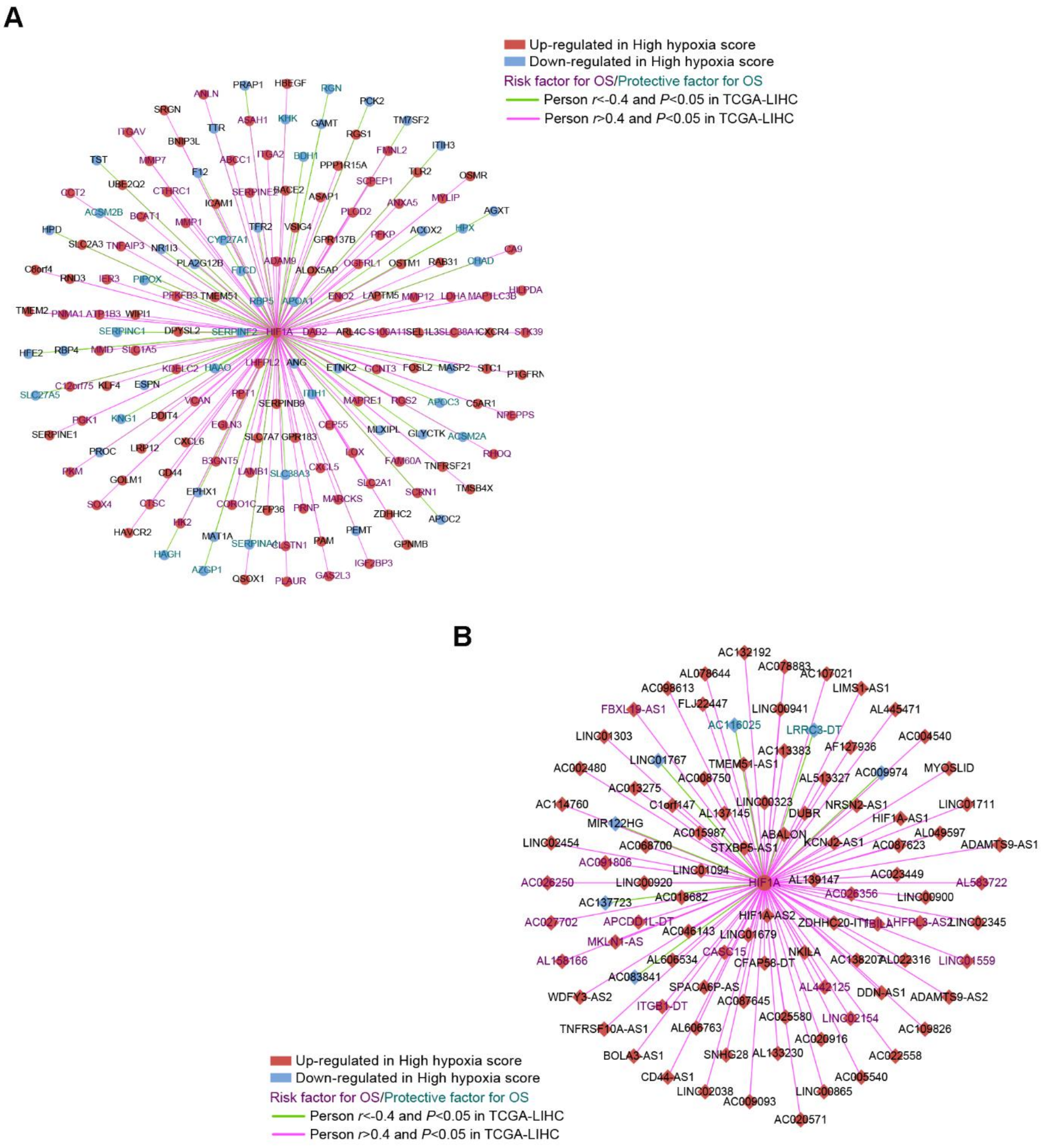

Supplementary Figure 4. DE-mRNAs and DE-lncRNAs correlated with hypoxia-inducible factor 1-alpha (HIF-1A) mRNA. The correlations were calculated by Pearson correlation analysis using TCGA-LIHC data. (**A**) DE-mRNAs correlated with HIF-1A mRNA. (**B**) DE-lncRNAs correlated with HIF-1A mRNA.

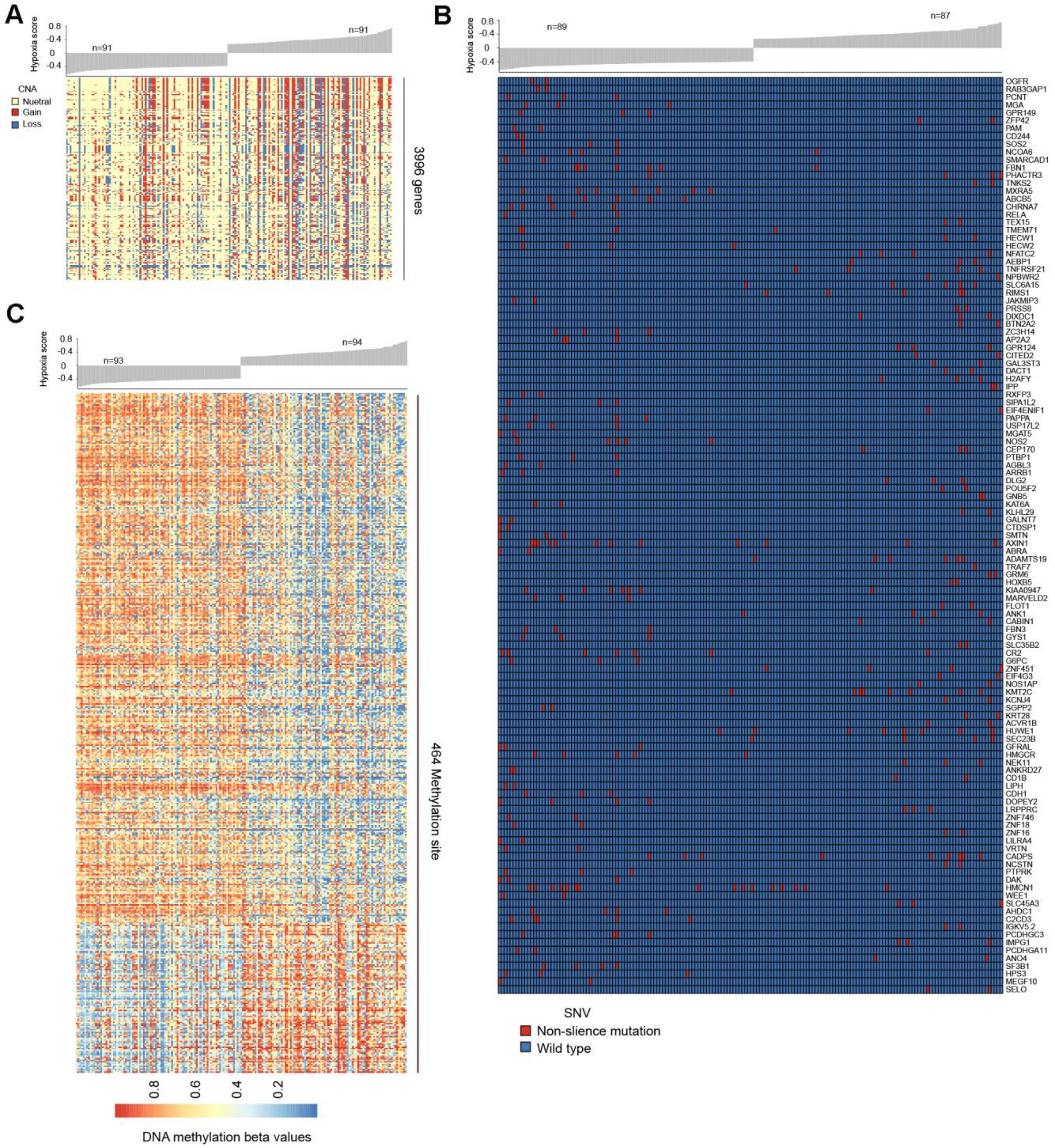

Supplementary Figure 5. Overview of genomic alterations and DNA methylation changes in groups with high hypoxia scores and low hypoxia scores. (**A**) Difference in the proportions of copy-number aberrations (CNAs) between the high hypoxia score group and the low hypoxia score group. (**B**) Proportions of single-nucleotide variants (SNVs) in 172 genes distributed in the low hypoxia score group and the high hypoxia score group. (**C**) A total of 464 loci with different methylation levels in the high hypoxia score group and the low hypoxia score group.

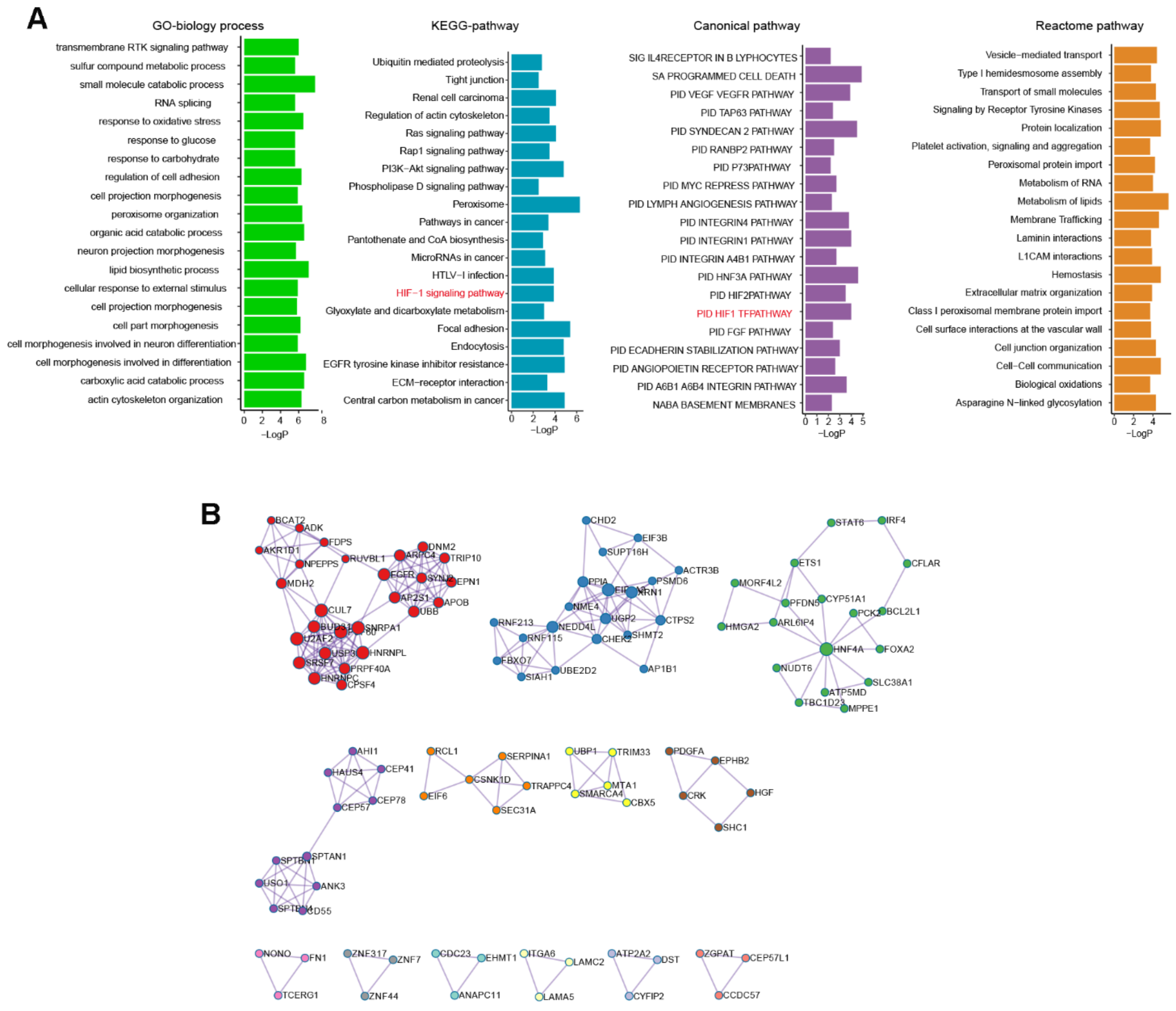

MCODE_1	R-HSA-72163	mRNA Splicing - Major Pathway	-14.4
MCODE_1	R-HSA-72172	mRNA Splicing	-14.2
MCODE_1	R-HSA-8856828	Clathrin-mediated endocytosis	-13.4
MCODE_2	R-HSA-983168	Antigen processing: Ubiquitination & Proteasome degradation	-8.5
MCODE_2	R-HSA-1280218	Adaptive Immune System	-8.5
MCODE_2	GO:0000209	protein polyubiquitination	-8.3
MCODE_3	M28	PID IL4 2PATHWAY	-6.8
MCODE_3	GO:0035690	cellular response to drug	-5.2
MCODE_3	GO:0048732	gland development	-4.9
MCODE_4	R-HSA-6807878	COPI-mediated anterograde transport	-11.7
MCODE_4	R-HSA-199977	ER to Golgi Anterograde Transport	-10.6
MCODE_4	GO:0140056	organelle localization by membrane tethering	-10.3
MCODE_5	GO:0048208	COPII vesicle coating	-9.1
MCODE_5	GO:0048207	vesicle targeting, rough ER to cis-Golgi	-9.1
MCODE_5	R-HSA-204005	COPII-mediated vesicle transport	-9.1
MCODE_7	GO:0043405	regulation of MAP kinase activity	-9.2
MCODE_7	GO:0071900	regulation of protein serine/threonine kinase activity	-8.3
MCODE_7	GO:0033674	positive regulation of kinase activity	-8.0
MCODE_10	R-HSA-8853884	Transcriptional Regulation by VENTX	-8.4
MCODE_10	R-HSA-2559582	Senescence-Associated Secretory Phenotype (SASP)	-7.0
MCODE_10	R-HSA-2559583	Cellular Senescence	-6.3
MCODE_11	M158	PID INTEGRIN4 PATHWAY	-10.2
MCODE_11	R-HSA-3000157	Laminin interactions	-8.8
MCODE_11	M239	PID A6B1 A6B4 INTEGRIN PATHWAY	-8.2

Supplementary Figure 6. Functional enrichment analysis of biological processes and pathways for 681 mRNAs involving differential AS events. (**A**) A total of 681 mRNAs are involved in AS events with differences in occurrences between the high hypoxia score group and the low hypoxia score group. The top 20 (sorted by P-value) enrichment analysis results for GO biological processes and enrichment analysis results for pathways from 3 different data sources are displayed. (**B**) The protein-protein interaction (PPI) enrichment network of the translation products of the 681 mRNAs were constructed using the MCODE algorithm.

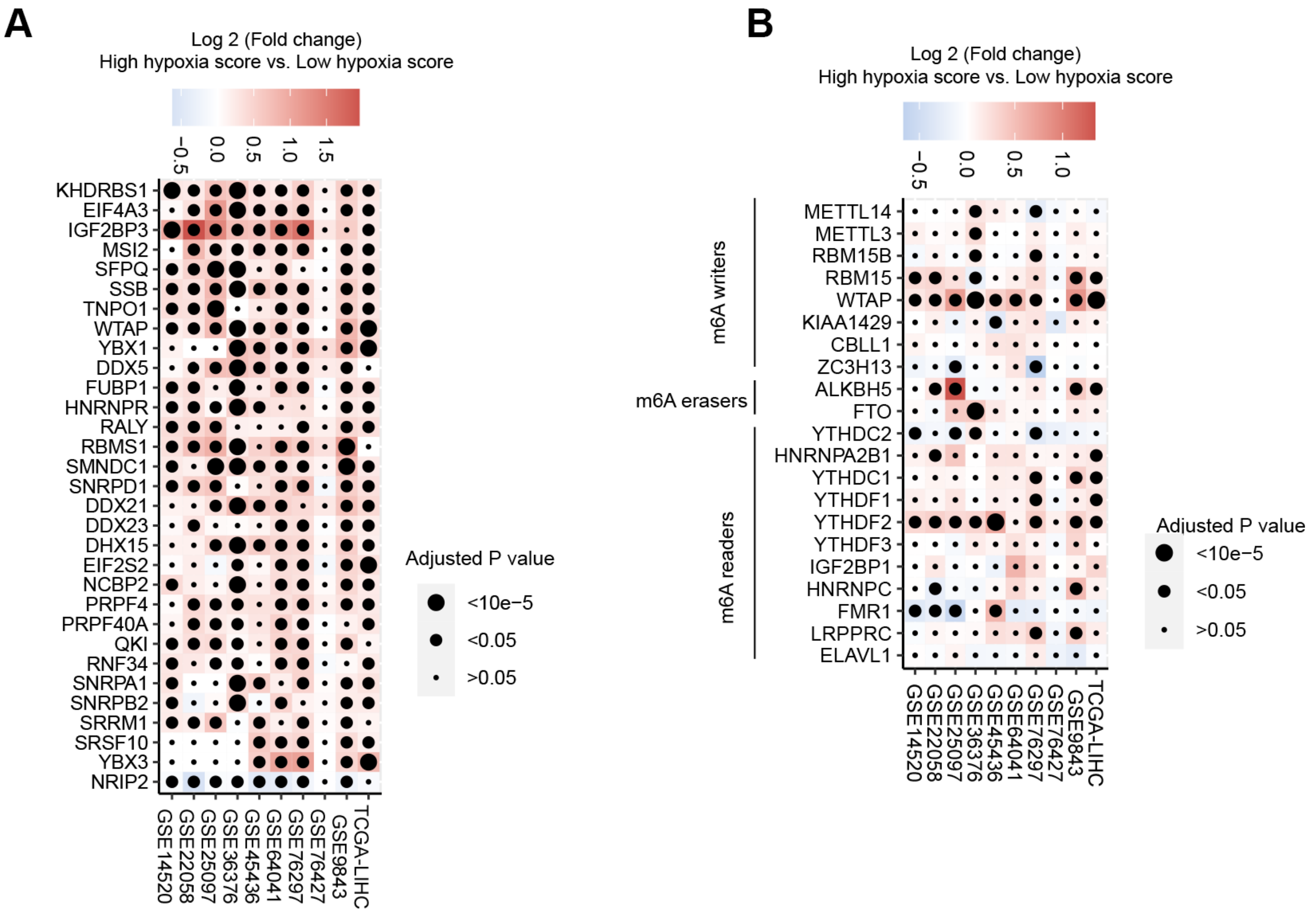

Supplementary Figure 7. The expression of 30 splicing factors and 21 N6-methyladenosine (m6A) regulators in the high hypoxia score group and the low hypoxia score group. (**A**) Differences in the expression of 30 splicing factors between the high hypoxia score groups and the low hypoxia score groups in 10 HCC datasets. (**B**) Differences in the expression of 21 m6A regulators between the high hypoxia score groups and the low hypoxia score groups in 10 HCC datasets.

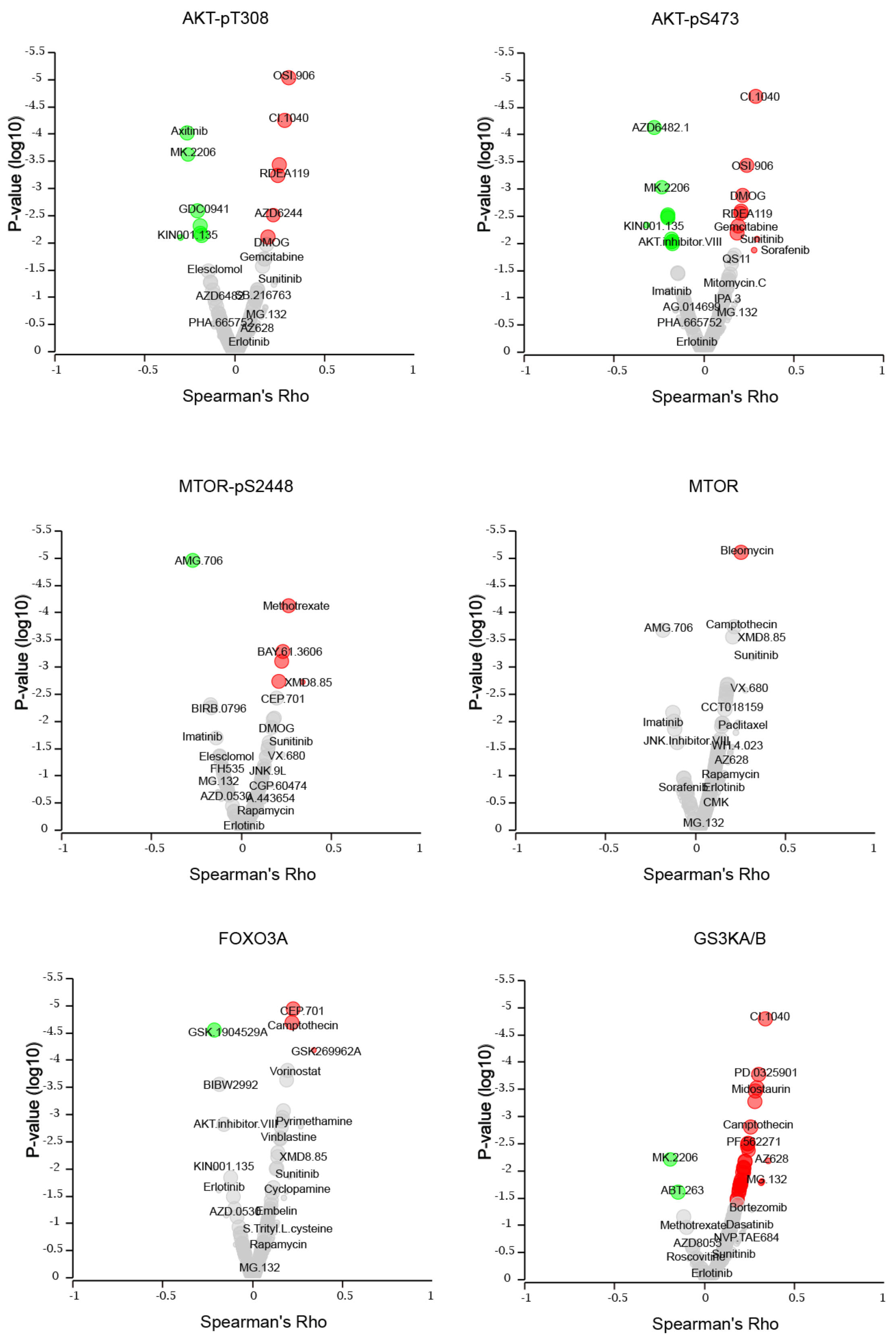

Supplementary Figure 8. Spearman's correlation coefficients for proteins with significantly different expression in the high hypoxia score group and the low hypoxia score group and commonly used anticancer drugs. The data were obtained from the MD Anderson Cell Lines Project and the Genomics of Drug Sensitivity in Cancer database.
