# Supplementary material for: Hypoxia associated multi-omics molecular landscape of tumor tissue in patients with hepatocellular carcinoma
Source: Aging (Albany NY). 2021 Mar 10;13(5):6525–53. doi: 10.18632/aging.202723 (PMC7993683; doi:10.18632/aging.202723)
Supplement: Supplementary Table 1 [file aging-13-202723-s002.pdf]

Supplementary Table 1. The information for elements of 21-gene hypoxia signature.

Gene symbol	Description	Gene Location	Protein Function (Protein Atlas)	Refseq
CA9	carbonic anhydrase 9	chr9:35673928-35681157	Enzymes/ENZYME proteins/Lyases	NM_001216
PFKFB4	6-phosphofructo-2-kinase/fructose-2,6-biphosphatase 4	chr3:48517684-48556803;chr3:48517684-48561129	Enzymes/ENZYME proteins/{Hydrolases,Transferases}	NM_001317134
HILPDA	hypoxia inducible lipid droplet associated	chr7:128455878-128458418;chr7:128455830-128458418	N/A	NM_001098786
BNIP3L	BCL2 interacting protein 3 like	chr8:26390413-26413127;chr8:26383054-26413127	Transporters/Transporter channels and pores	NM_004331
SLC2A3	solute carrier family 2 member 3	chr12:7919230-7936187	Transporters/Electrochemical Potential-driven transporters	NM_006931
PLIN2	perilipin 2	chr9:19115761-19127606;chr9:19115761-19127492	N/A	NM_001122
KDM3A	lysine demethylase 3A	chr2:86441461-86492716;chr2:86441371-86492716	N/A	NM_001146688
INSIG2	insulin induced gene 2	chr2:118088418-118110031;chr2:118088550-118110031;chr2:118088471-118110997	N/A	NM_001321329
EGLN3	egl-9 family hypoxia inducible factor 3	chr14:33924227-33951074	Enzymes/ENZYME proteins/Oxidoreductases	NM_022073
GDF15	growth differentiation factor 15	chr19:18386158-18389176	N/A	NM_004864
PTPRH	protein tyrosine phosphatase receptor type H	chr19:55181247-55209501	Enzymes/ENZYME proteins/Hydrolases	NM_002842
HCAR3	hydroxycarboxylic acid receptor 3	chr12:122714756-122716811	G-protein coupled receptors/GPCRs excl olfactory receptors	NM_006018
SPAG4	sperm associated antigen 4	chr20:35615884-35621094;chr20:35615829-35621094	N/A	NM_001317931
WSB1	WD repeat and SOCS box containing 1	chr17:27294114-27315926	N/A	NM_001348350
TMEM45A	transmembrane protein 45A	chr3:100492619-100577444	N/A	NM_018004
ADM	adrenomedullin	chr11:10305073-10307397	N/A	NM_001124
BNIP3	BCL2 interacting protein 3	chr10:131967683-131982013	Transporters/Transporter channels and pores	NM_004052
JUN	Jun proto-oncogene, AP-1 transcription factor subunit	chr1:58780791-58784047	Transcription factors/Basic domains	NM_002228
SMAD3	SMAD family member 3	chr15:67065602-67195167;chr15:67166155-67195195;chr15:67138021-67195195;chr15:67125716-67195195	Transcription factors	NM_005902
HK2	hexokinase 2	chr2:74834127-74893358;chr2:74835170-74893358	Enzymes/ENZYME proteins/Transferases	NM_000189
GYS1	glycogen synthase 1	chr19:48968130-48993309	Enzymes/ENZYME proteins/Transferases	NM_002103
