# Supplementary material for: Hypoxia associated multi-omics molecular landscape of tumor tissue in patients with hepatocellular carcinoma
Source: Aging (Albany NY). 2021 Mar 10;13(5):6525–53. doi: 10.18632/aging.202723 (PMC7993683; doi:10.18632/aging.202723)
Supplement: Supplementary Tables 5 and 6 [file aging-13-202723-s006.pdf]

Supplementary Table 5. Annotation for MCODE of protein-protein interaction enrichment network for high frequency DE-mRNAs.

Network	Annotation
Input ID	GO:0032787|monocarboxylic acid metabolic process|-24.6;GO:0009611|response to wounding|-23.6;hsa04610|Complement and coagulation cascades|-22.6
Input ID_MCODE_ALL	GO:0032787|monocarboxylic acid metabolic process|-16.6;hsa01230|Biosynthesis of amino acids|-16.0;GO:0044282|small molecule catabolic process|-15.0
Input ID_SUB2_MCODE_1	R-HSA-211897|Cytochrome P450 - arranged by substrate type|-23.3;R-HSA-211945|Phase I - Functionalization of compounds|-21.4;R-HSA-211859|Biological oxidations|-18.4
Input ID_SUB1_MCODE_2	R-HSA-381426|Regulation of Insulin-like Growth Factor (IGF) transport and uptake by Insulin-like Growth Factor Bi|-5.1;R-HSA-71387|Metabolism of carbohydrates|-4.0;GO:0031589|cell-substrate adhesion|-3.8
Input ID_SUB1_MCODE_3	R-HSA-375276|Peptide ligand-binding receptors|-14.6;R-HSA-373076|Class A/1 (Rhodopsin-like receptors)|-13.0;R-HSA-418594|G alpha (i) signalling events|-12.4
Input ID_SUB1_MCODE_4	hsa01230|Biosynthesis of amino acids|-8.5;GO:0044282|small molecule catabolic process|-7.4;hsa00270|Cysteine and methionine metabolism|-6.7
Input ID_SUB1_MCODE_5	GO:0046364|monosaccharide biosynthetic process|-14.4;hsa01200|Carbon metabolism|-14.0;GO:0016051|carbohydrate biosynthetic process|-12.3
Input ID_SUB1_MCODE_6	R-HSA-5686938|Regulation of TLR by endogenous ligand|-8.8;M264|PID TOLL ENDOGENOUS PATHWAY|-8.4;R-HSA-6803157|Antimicrobial peptides|-6.6
Input ID_SUB1_MCODE_7	M166|PID ATF2 PATHWAY|-7.9;GO:0009991|response to extracellular stimulus|-5.0;R-HSA-2262752|Cellular responses to stress|-4.8

Supplementary Table 6. Survival analysis for Differential expressed miRNAs in TCGA-LIHC.

ID	log-rank P value	factor	HR	up95	low95
hsa-miR-101-3p	0.002255	protect	0.578932	0.820239	0.408615
hsa-miR-139-5p	2.43E-07	protect	0.394906	0.56083	0.278072
hsa-miR-511-5p	0.391565	protect	1.163998	1.648429	0.821928
hsa-let-7c-3p	0.509074	protect	0.88955	1.259684	0.628172
hsa-miR-125b-2-3p	0.061228	protect	0.718071	1.017179	0.506917
hsa-miR-34c-5p	0.52498	risk	1.11925	1.584939	0.79039
hsa-miR-378c	0.277779	protect	0.82486	1.168134	0.582463
hsa-miR-5589-5p	0.229327	protect	0.809008	1.14691	0.570659
hsa-miR-99a-5p	0.008725	protect	0.627161	0.888743	0.442571
hsa-miR-34c-3p	0.978891	protect	1.004682	1.422972	0.709351
hsa-miR-5589-3p	0.420898	protect	0.867101	1.228034	0.61225
hsa-miR-877-5p	0.016982	risk	1.529861	2.167074	1.080017
hsa-miR-671-5p	0.295045	risk	1.202313	1.702882	0.848888
hsa-miR-378a-3p	0.196099	risk	1.257156	1.781223	0.887279
hsa-miR-301a-3p	0.008197	risk	1.595768	2.262316	1.125605
hsa-miR-187-3p	0.538865	risk	1.11492	1.579794	0.786841
hsa-miR-138-5p	0.650067	risk	0.921018	1.311126	0.646981
hsa-miR-4524a-3p	0.399592	protect	0.861413	1.219765	0.60834
hsa-miR-223-3p	0.951984	risk	1.010698	1.431194	0.713747
hsa-let-7a-2-3p	0.679079	protect	0.929348	1.315997	0.656299
hsa-miR-375-3p	0.456514	protect	0.876533	1.2419	0.618658
hsa-miR-223-5p	0.742728	protect	0.943599	1.336783	0.666061
hsa-miR-5588-3p	0.012826	protect	0.640822	0.907489	0.452515
hsa-miR-3189-3p	0.023056	risk	1.490708	2.122278	1.047088
hsa-miR-3680-3p	0.127824	risk	1.30919	1.855126	0.923915
hsa-miR-17-3p	0.083783	risk	0.733445	1.038702	0.517898
hsa-miR-194-3p	0.165805	protect	0.782338	1.108308	0.55224
hsa-miR-135b-5p	0.072067	risk	1.376916	1.949932	0.972289
hsa-miR-7112-3p	0.515253	risk	1.123438	1.602959	0.787365
hsa-miR-205-5p	0.171105	risk	1.272456	1.804744	0.89716
hsa-miR-100-5p	0.003693	protect	0.598643	0.849333	0.421947
hsa-miR-146a-5p	0.745306	protect	1.059352	1.500225	0.748039
hsa-miR-24-2-5p	0.081881	risk	1.362826	1.9299	0.962379
hsa-miR-885-5p	0.169666	protect	0.784611	1.112856	0.553184
hsa-miR-885-3p	0.603559	protect	1.09687	1.554033	0.774195
hsa-miR-1262	0.014415	risk	1.539902	2.184093	1.085713
hsa-miR-582-5p	0.388832	risk	1.164231	1.650574	0.821189
hsa-miR-582-3p	0.022381	risk	1.493008	2.120278	1.051312
hsa-miR-210-5p	0.024344	risk	1.491626	2.113712	1.052627
hsa-miR-217-5p	0.826759	protect	1.039643	1.472817	0.733871
hsa-miR-625-3p	0.578341	protect	1.103574	1.562673	0.779354
hsa-miR-29b-1-5p	0.870187	risk	1.029333	1.457906	0.726746
hsa-miR-21-3p	0.060608	risk	1.393654	1.97479	0.983533
hsa-miR-561-5p	0.0075	risk	1.62099	2.40279	1.093566
hsa-miR-541-3p	0.568232	protect	1.106522	1.566843	0.781438
hsa-miR-216b-3p	0.46815	risk	1.150479	1.701683	0.77782
hsa-miR-1224-5p	0.886944	risk	1.025507	1.452126	0.724225
hsa-miR-5588-5p	0.089422	protect	0.739409	1.047195	0.522086
hsa-miR-2355-3p	0.008464	risk	1.595746	2.261333	1.126063
hsa-miR-216a-3p	0.335209	risk	1.186604	1.680295	0.837965
hsa-miR-4449	0.844205	protect	1.035457	1.466219	0.731249
hsa-miR-653-5p	0.577505	protect	1.103923	1.563284	0.779543

hsa-miR-194-5p	0.004083	protect	0.601321	0.851918	0.424439
hsa-miR-548v	0.174238	risk	1.271901	1.802342	0.897572
hsa-miR-141-3p	0.535513	risk	1.115914	1.580405	0.787941
hsa-miR-216a-5p	0.342731	protect	1.18372	1.676152	0.835959
hsa-miR-155-5p	0.45926	risk	1.140173	1.614518	0.805191
hsa-miR-216b-5p	0.131044	protect	1.308972	1.853506	0.924414
hsa-miR-155-3p	0.832344	risk	1.038098	1.470129	0.733029
hsa-miR-210-3p	0.000975	risk	1.791011	2.541255	1.262258
hsa-miR-629-5p	0.040936	protect	1.442479	2.042647	1.018651
hsa-miR-642a-5p	0.690464	risk	1.073046	1.519633	0.757701
hsa-miR-200c-3p	0.038228	risk	1.443662	2.045728	1.018786
