# Supplementary material for: Hypoxia associated multi-omics molecular landscape of tumor tissue in patients with hepatocellular carcinoma
Source: Aging (Albany NY). 2021 Mar 10;13(5):6525–53. doi: 10.18632/aging.202723 (PMC7993683; doi:10.18632/aging.202723)
Supplement: Supplementary Table 11 [file aging-13-202723-s011.pdf]

Supplementary Table 11. DNA methylation changes of 30 up-regulated genes in high hypoxia score groups.

site	Gene	DNA methylation level	Position_to_TSS
cg23174662	HIF1A	reduced	3587;3824;3824;1742;3851;469;3851;1742
cg04293307	AXIN2	reduced	4185;1267;2864;4185;83571
cg06075789	ZFP36	reduced	.
cg17382541	MYADM	reduced	1523;1227;-299;-299;1361;-299;-299
cg09442403	TM4SF1	reduced	6355;4202;6182;6232
cg03707168	PPP1R15A	reduced	3477;1551
cg04797170	TMSB10	reduced	252;252
cg12232146	PHLDA1	reduced	29859
cg12457415	CLIC1	reduced	1488;2234;1483;4679;1461
cg26403843	RNF145	reduced	750;2475;2977;997;2715;558;1056;2522
cg05374271	RGS2	reduced	718;717;720;616
cg27270412	NRP1	reduced	2859;1119;1119;1239;979;1502;1233;1502;1675
cg16643088	CSRNP1	reduced	6324;7311
cg13275603	ENC1	reduced	9763;601;9763;8988;9763
cg22502206	ANXA2	reduced	-641;-640;-640;-583;4257;-640;-640;-653;-640;-638;-652;-640;-593;-662;-640;-640;-648;-638;-625;-652;116;-642;-658;-1288;-640;-640;-638;-640;-640;-640;-640
cg09055236	TTYH3	reduced	1611;1406
cg05132999	TTC39A	reduced	-1141;32;-752;-1112;13802;-367;13802;-750;13802;1660
cg10523105	PNMA1	reduced	2100
cg15460348	SPP1	reduced	89;42;68;42;-1124;39;42;42;41;42;106
cg03359362	SLC1A5	reduced	-1462;-1462;989;2241;-1448
cg05117638	MARCKS	reduced	18616;1056
cg11230435	MAP3K8	reduced	1507;1200;779;1303;1423
cg27505627	BAIAP2L2	reduced	64;89
cg00937742	LYPD1	reduced	-146
cg22679626	TMSB4X	reduced	.
cg19913563	IER3	reduced	.
cg22249612	CD63	reduced	1520;950;1960;200;-1344;-641;-592;2007;1300;115;947;-572;1277;782;649;-1404
cg04860291	TKT	reduced	3379;3388;3366;3388;3397;3340;3353;3418
cg12586150	SERPINB1	reduced	1449;1305;1291;1402
cg24147596	ARL14	reduced	472
